# Nontargeted Metabolomics as a Screening Tool for Estimating Bioactive Metabolites in the Extracts of 50 Indigenous Korean Plants

**DOI:** 10.3390/metabo11090585

**Published:** 2021-08-30

**Authors:** Se Rin Choi, Mee Youn Lee, Seung A Kim, Jieun Oh, Da Won Hyun, Sarah Lee, Byoung-Hee Lee, Jae Youl Cho, Choong Hwan Lee

**Affiliations:** 1 Department of Bioscience and Biotechonology, Konkuk University, Seoul 05029, Korea; csr0701@gmail.com (S.R.C.); kkamlice@hanmail.net (M.Y.L.); 2 Department of Integrative Biotechnology, Sungkyunkwan University, Suwon 16419, Korea; seung-a26@naver.com (S.A.K.); martia96@gmail.com (J.O.); jaecho67@gmail.com (J.Y.C.); 3 National Institute of Biological Resources, Environmental Research Complex, Incheon 22689, Korea; dwhyun183@korea.kr (D.W.H.); lsr57@korea.kr (S.L.); dpt510@korea.kr (B.-H.L.); 4 Research Institute for Bioactive-Metabolome Network, Konkuk University, Seoul 05029, Korea

**Keywords:** indigenous plant, metabolite profiling, UHPLC-LTQ-Orbitrap-MS/MS, anti-inflammatory activity, antioxidant activity

## Abstract

Many indigenous Korean plants have been used in medicinal preparations and health-promoting foods. These plant species contain beneficial metabolites with various bioactivities, such as antioxidant and anti-inflammatory activities. Herein, we suggest a new screening strategy using metabolomics to explore the bioactive compounds in 50 Korean plants. Secondary metabolites were analyzed using UHPLC-LTQ-Orbitrap-MS/MS. The plant extracts were subjected to antioxidant and anti-inflammatory assays. We identified metabolites that contributed to bioactivities according to the results of bioassays and multivariate analyses. Using Pearson’s correlation, phenolics (e.g., casuarictin, 3-O-methylellagic acid) showed positive correlation with antioxidant activity, while biflavonoids (e.g., amentoflavone, rosbustaflavone) were correlated with nitric oxide (NO) inhibition activity. To compensate for the limitation of this new strategy, we further validated these by investigating three parts (branches, fruits, leaves) of *Platycladus orientalis* which showed high activities on both bioassays. Unlike the above observation, we identified significantly different metabolites from different parts, which was not the results of bioassays. In these validation steps, interestingly, biflavonoids (e.g., robustaflavone, sciadopitysin) contributed to both activities in *P. orientalis*. The findings of this work suggest that new strategy could be more beneficial in the identification of bioactive plant species as well as that of their corresponding bioactive compounds that impart the bioactivity.

## 1. Introduction

Korea has a wide diversity of plant species due to its various geo-climatic conditions. Many indigenous Korean plants have been used as health-promoting foods [1], antioxidant agents [2], and raw materials of cosmetics [3]. The use of plant species has been related to secondary metabolites, such as flavonoids, saponins, terpenoids, and alkaloids in plants. They have various bioactivities, such as antioxidant, anti-inflammatory, antimicrobial, and anticancer activities [4,5,6,7]. For this reason, secondary metabolites have been used as important ingredients of traditional medicine and as food additives for centuries [8,9]. To date, they have been widely used as valuable compounds, in pharmaceuticals, cosmetics, and more recently, nutraceuticals [10,11]. Thus, to utilize indigenous Korean plant species more effectively, profiling their bioactive compounds is necessary. The screening of the relevant bioactive plants can be accomplished by comparing the differences in the bioactivities such as antioxidant activity of various plant species and exploring the secondary metabolites of the indigenous species.

Considering the promising benefits of the indigenous plants on human health and of their bioactive compounds that are correlated with health benefits, we compared the bioactivities of 50 indigenous Korean plants. The underlying mechanisms of many metabolites, such as flavonoids, that impart their bioactivity are thought to be mediated by their free radical scavenging activities [12]. Many biological processes are mediated by nitric oxide (NO), a short-lived free radical. NO can enhance the bactericidal and tumoricidal activities of activated macrophages [13,14], but when produced in excess, can potentially lead to tissue damage and the activation of pro-inflammatory mediators [15]. Reactive oxygen species (ROS) are produced by cellular metabolism, and since they can cause oxidative damage to lipids, nucleic acids, and proteins, they are highly toxic and are involved in the diagnosis of many chronic diseases. Additionally, Seifried et al. [16] have shown that overproduction of free radical molecules as ROS and reactive nitrogen species (RNS) are linked with a number of inflammatory disorders. Furthermore, ROS is potent activator of NF-κB signaling pathway; indirectly promoting the iNOS expression and NO synthesis [17]. Thus, it is necessary to consider the inhibition of free radical scavenging as an important therapeutic consideration the in development of anti-inflammatory agents. Although humans have an internal system of antioxidants in the body, exogenous antioxidants are recommended [18]. Antioxidants can be natural or synthetic, but due to their toxic and carcinogenic effects, synthetic antioxidants are being replaced with natural antioxidants [19]. The increase in dietary antioxidant intakes may help to support the limiting antioxidant concentrate and also promote the normal functioning of physiological systems [20]. The potential of the extracts from indigenous Korean plants to scavenge these free radicals and modulate inflammatory reactions has been demonstrated [21,22,23]. Additionally, many researchers have used the antioxidant activity assay to reveal the beneficial effect of different plant extracts [24]. Therefore, to utilize these extracts in a proper and effective way, it is important to find bioactive compounds that contribute to NO inhibitory and antioxidant activities. We assayed the NO production inhibitory and antioxidant activities of the extracts of 50 indigenous Korean plants. Additionally, we correlated the results of these assays with their metabolites to identify the bioactive compounds in 50 indigenous Korean plant samples using a high-throughput screening method.

From this perspective, metabolomics enables an unbiased, high-throughput screening through chromatographic separation, high-resolution mass spectrometry (MS), and enhances detection sensitivity [25]. This metabolite characterization is a powerful tool for the comprehensive identification and quantification of metabolites in plants. Many studies have identified beneficial secondary compounds in plants and reported their bioactivities, such as antioxidant and anti-inflammatory activities [26,27,28]. In addition, metabolomics has been used as a chemotaxonomic tool for the classification of plant species [29]. Chemotaxonomic plant classification has been used to classify plant species according to their phylogenetic genus [30]. Some studies have used this classification to reveal the relationship between the differences in the active metabolites and bioactivities of diverse plant species [31,32,33]. However, only a few studies have attempted to identify bioactive compounds in diverse plant species based on bioactivity assays. 

Herein, we aimed (1) to suggest a new screening strategy using a multi-parallel metabolomic-cum-bioassay-guided approach to identify bioactive compounds in 50 indigenous plant extracts, and (2) to validate these bioactive compounds by profiling the metabolites found in the different parts (fruit, branch, and leaf) of *Platycladus orientalis* which contributed to anti-inflammatory and antioxidant activities. This validation technique solves the correlation bias caused by the screening strategy that uses the multi-parallel metabolomic-cum-bioassay-guided approach.

## 2. Results and Discussion

### 2.1. Bioactivities of Indigenous Korean Plant Species

To select superior plant extracts based on their bioactivity, NO inhibitory and antioxidant activity was measured using 2,2-azinobis (3-ethylbenzothiazoline-6-sulfonic acid (ABTS) and 2,2-diphenyl-1-picrylhydrazyl (DPPH) assays. The bioactivity of plant extracts is mainly due to the diverse composition of secondary metabolites that fulfill multiple ecological roles among plant species [34]. The inhibitory activity of the extracts on NO production by activated RAW 264.7 cell lines is presented in Figure S1. Seven plant species showed high inhibitory activity on NO production, namely *Commelina communis, Elaeagnus umbellata, Eleutherococcus sessiliflorus, P. orientalis, Pinus densiflora, Zanthoxylum schinifolium,* and *Sophora flavescens*, as determined by the 3- (4,5-dimethylthiazol-2-yl)-2,5-diphenyltetrazolium bromide (MTT) assay. The results are in line with those of previous studies wherein plant species were revealed to have anti-inflammatory effects [35,36,37,38,39,40]. The antioxidant activity of 50 samples (Figure S2) is presented as the standard of Trolox equivalent antioxidant activity. There were variations in the antioxidant activity among the samples. *Platycladus orientalis, E. umbellata, Castanea crenata,* and *Machilus thumbergii* had the highest antioxidant activities among the 50 samples. Similarly, these four species also showed antioxidant activity in other studies [41,42,43,44]. 

### 2.2. Metabolite Profiling of the Bioactivities of 50 Indigenous Korean Plants

**Figure 1:**
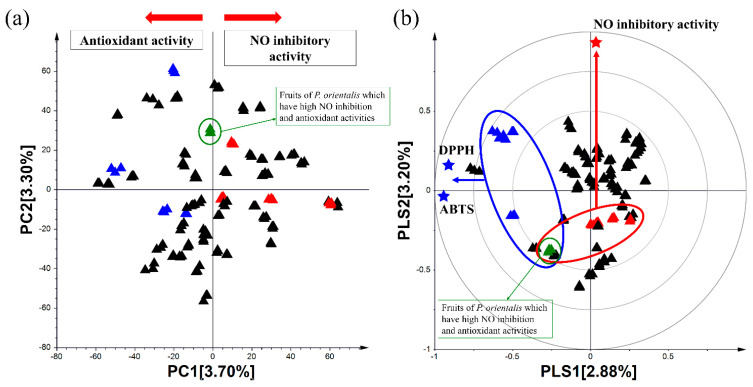
(**a**) PCA score plot; (**b**) PLS biplot derived from the UHPLC-LTQ-Orbitrap-MS dataset for Table 1. indigenous plants. PLS biplot shows the correlation between the metabolite variations and the selected test bioactivities in the extracts of the 50 indigenous plants. The samples were analyzed using three analytical replicates for each sample. ▲: Plant species which have high antioxidant activities; ▲: Plant species which have high NO inhibition activity; ▲: Plant species which include in 50 indigenous plants.

The samples were also analyzed by UHPLC-LTQ-Orbitrap-MS/MS combined with multivariate statistical analysis. In principal component analysis (PCA), the 50 samples were clustered according to the results of their bioactivities (Figure 1a). Plant species with high NO inhibitory activity were found to be distinct from those with high antioxidant activities by PC1 (3.70%). Considering the clear variance defined by the PCA (Figure 1a) and the bioactivity analysis (Figures S1 and S2), a supervised multivariate data analysis (partial least squares (PLS)) was employed to organize and distinguish the samples according to their MS dataset. The correlation analysis of the bioactivities and metabolites of 50 samples was conducted by the setting the results of the bioactivity assays as Y variables and the MS dataset as the X variable (Figure 1b). Similar to the results of the PCA analysis, plant extracts with NO inhibitory and antioxidant activities are clustered in PLS model, respectively. The findings demonstrated that the metabolites of the plant extracts which showed superior NO inhibitory activity were strongly correlated with this activity. Likewise, the metabolites of the superior antioxidative extracts were related to their antioxidant activities. These results were also confirmed with the results of the bioassays. Interestingly, fruit of *P. orientalis* that showed high bioactivities in both assays were located in the middle of superior NO inhibitory and antioxidant extracts in PCA and PLS biplot (Figure 1).

**Figure 2:**
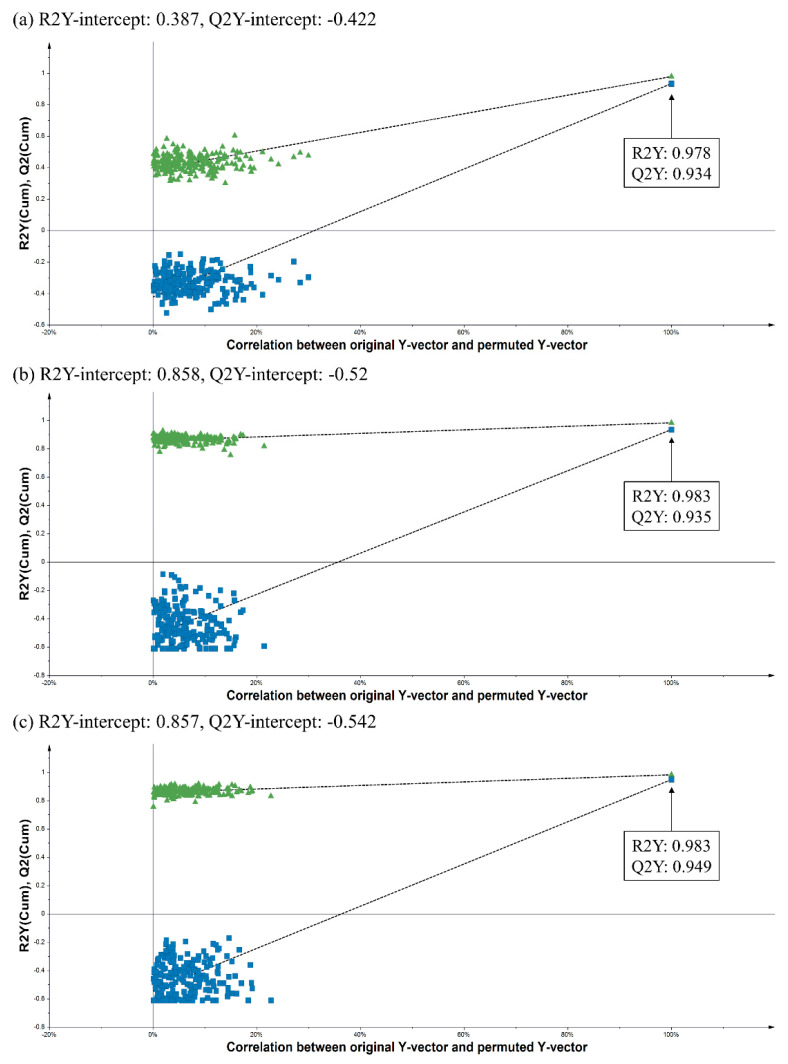
Permutation plots of the PLS model describing the R2 and Q2 Y-intercepts for NO inhibitory (**a**), ABTS (**b**), and DPPH (**c**) activities of 50 indigenous plant extracts. The PLS model was validated using 200 permutation tests to evaluate its goodness of fit and predictive power.

Variable importance to projections (VIP) values were acquired to measure the contribution of a variable to the model. A region of VIP > 1 indicates that the metabolites that can be distinguished as important for optimal PLS model performance [45]. Whereas, compounds with VIP values less than 1.0 might indicate that they have lower contribution to the bioactivities. Each VIP values of tentatively identified metabolites were shown in Tables S2 and S3. Additionally, a permutation test with 200 random permutations was performed to confirm the validity of the PLS biplot with the goodness of fit of several models and the predictive ability (R2/Q2) of the model (Figure 2). The PLS model has five components and showed a perfect goodness of fit where (R2Y (cum) > 0.9) and predictive quality (Q2 (cum) > 0.9). The permutation results of the NO inhibitory activity showed that Y-intercepts R^2^ and Q^2^ were 0.387 and −0.422; while those of the ABTS activity were 0.858 and −0.520, respectively, and the Y-intercept of R^2^ and Q^2^ of the DPPH activity were 0.857 and −0.542. These results indicated PLS was valid and not over fitting, exhibiting good predictive abilities [46].

In most plant metabolomics studies, researchers sorted variables from family, part or harvest time of their plant samples, not the bioactivity assay results, for profiling their metabolites [25,47,48]. Unlike this, we sorted many variables of 50 plant extracts based on their results of bioassays and multivariate statistical analysis which showed correlation with metabolites and bioactivities. After that, for finding the bioactive compound contributed to anti-inflammatory, we used the MS dataset of quality control (QC) sample which pooled all 50 sample extracts and the MS datasets of each of the five plants species that showed high NO inhibitory activities. Additionally, to identify metabolites that contributed to antioxidant activities, the MS datasets of QC and each of the five high antioxidative plant extracts were used. These secondary metabolites were tentatively characterized based on their retention time, mass spectra, mass fragment pattern, and elemental composition which were obtained from UHPLC-LTQ-Orbitrap-MS/MS datasets, as well as from published studies, and web databases such as NIST and MassBank. The obtained base peak chromatogram is illustrated in Figures S4 and S5. As a result of metabolite profiling according to bioactivity, 28 metabolites were identified as contributing to the superior NO inhibitory activity of the plant extracts, while 24 were identified as contributing to the high antioxidant activities (Tables S2 and S3).

**Figure 3:**
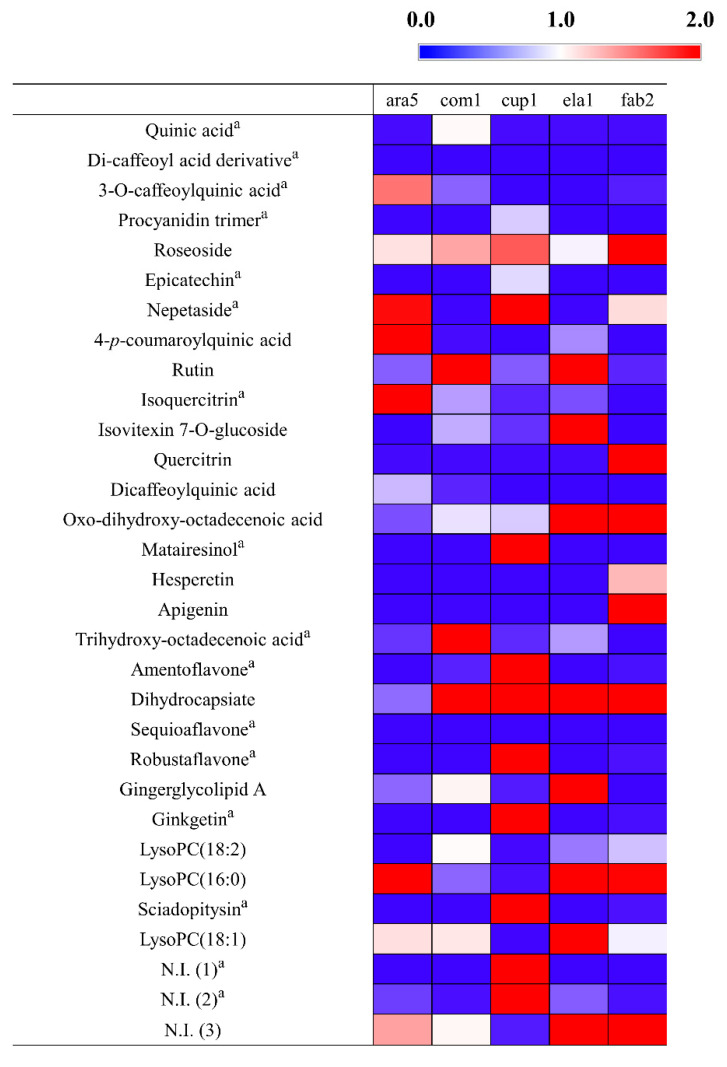
Heatmap analysis in plant species which have high NO inhibitory activities derived from UHPLC-LTQ-Orbitrap-MS/MS data. The heatmap indicates the relative contents in the secondary metabolites among the different fractions. ^a^ Metabolites that have a high contribution to the bioactivities were determined by PLS-biplot (VIP > 1.0, *p* < 0.05). N.I.: Non-identified metabolite.

**Figure 4:**
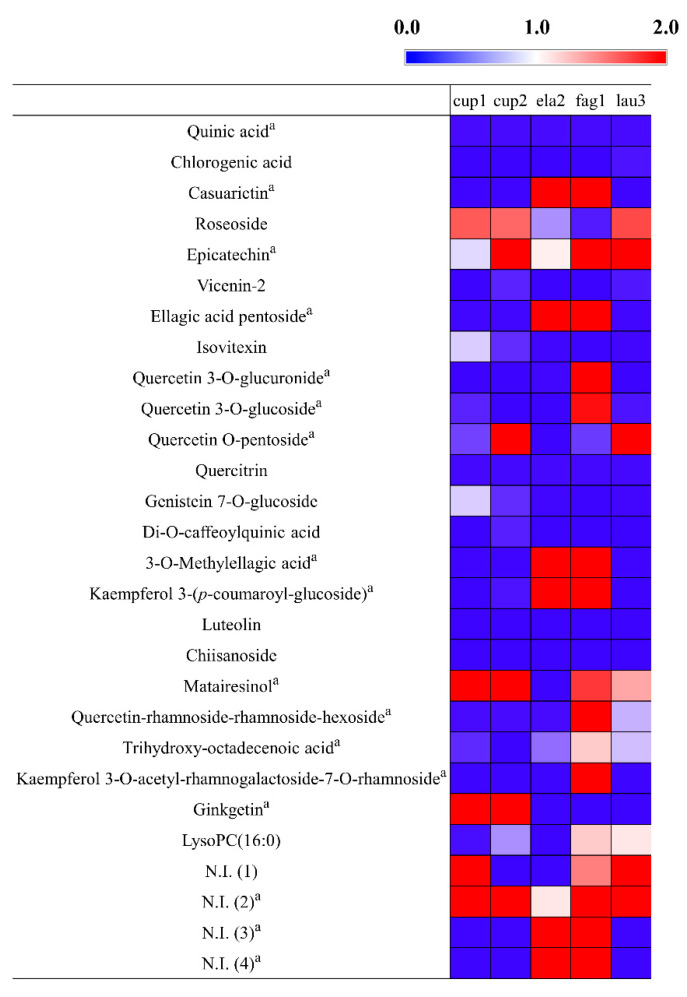
Heatmap analysis in plant species which have high antioxidant activities derived from UHPLC-LTQ-Orbitrap-MS/MS data. The heatmap indicates the relative contents in the secondary metabolites among the different fractions. ^a^ Metabolites that have high contribution to the bioactivities were determined by PLS-biplot (VIP > 1.0, *p <* 0.05). N.I.: Non-identified metabolite.

The relative levels of the secondary metabolites among the 50 samples were visualized using a heat map representation (Figures S7 and S8). The relative metabolites contents of the 5 plant extracts which showed high NO inhibitory were shown in Figure 3. Among the superior NO inhibitory plant extracts, most of the secondary metabolites had a higher relative abundance in the fruit of *P. orientalis*. The flavonoids in this plant have been reported to have a significant anti-inflammatory effect in lipopolysaccharide-induced macrophage cells [49]. Additionally, the diterpenes of *P. orientalis* have anti-inflammatory activities [50]. Meanwhile, the relative levels of secondary metabolites in the five superior antioxidant plant extracts was shown in Figure 4. The branches of *E. umbellata* and the fruit of *C. crenata* had relatively higher contents of metabolites. *E. umbellata* is a rich source of bioactive compounds, such as phenolic acids and flavonoids [51]. Tuyen et al. [43] revealed that *C. crenata* contains several phenolic compounds and provides promising antioxidant capacities.

### 2.3. Bioactivity Correlations for the Secondary Metabolites of Indigenous Korean Plants

**Figure 5:**
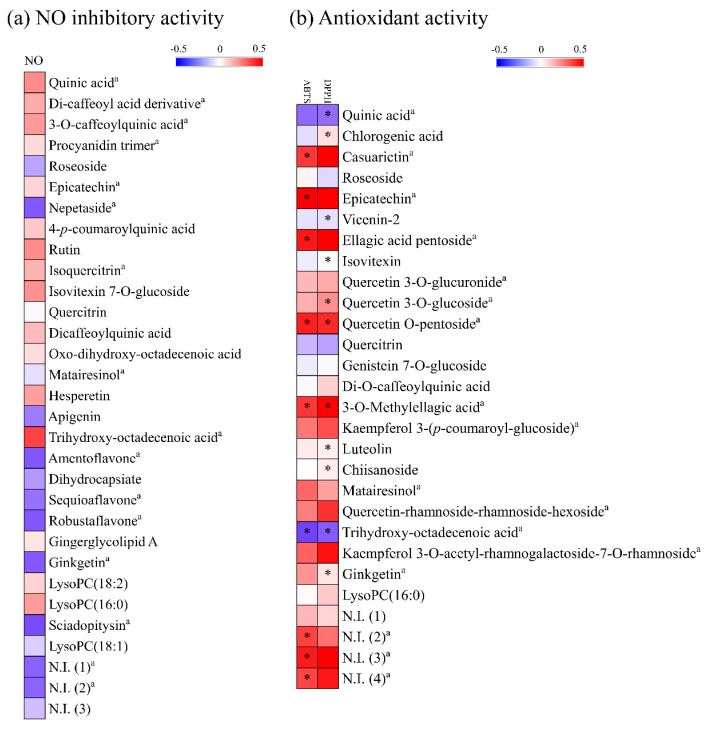
Correlation map between the relative abundance of the secondary metabolites and (**a**) NO production inhibitory activity and (**b**) antioxidant (ABTS, DPPH) activity of the extracts of 50 indigenous plants. Each square indicates Pearson’s correlation coefficient values (*r*). Red and blue represent positive (0 < *r* < 0.5) and negative (−0.5 < *r* < 0) correlations, respectively. * FDR-adjusted *p*-value < 0.05; ^a^ Metabolites that have a high contribution to the bioactivities were determined by PLS-biplot (VIP > 1.0). N.I.: Non-identified metabolites.

To visualize the correlation of the metabolites with NO inhibition and antioxidant activities, Pearson’s correlation test was used to construct a correlation map (Figure 5). *P* values were adjusted using Benjamini Hochberg false discovery rate (FDR), and only metabolites with an adjusted *P* value < 0.05 were considered statistically significant. As shown in Figure 5A, nepetaside, amentoflavone, sequoiaflavone, robustaflavone, ginkgetin, and sciadopitysin contributed to NO inhibitory activity. Interestingly, biflavonoids, such as amentoflavone and sequoiaflavone, showed significantly negative correlations with NO inhibitory activity. Biflavonoids are dimers of flavonoids linked to each other by C-C or C-O-C bond link-age [52]. Some studies have shown that biflavonoids inhibit NO production [53,54]. In addition to NO inhibition, they exhibit various anti-inflammatory activities [55]. As shown in Figure 5B, casuarictin, epicatechin, ellagic acid pentoside, 3-*O*-methylellagic acid, kaempferol 3- (*p*-coumaroyl-glucoside), and matairesinol were positively correlated with antioxidant activity.

Since multivariate analysis and identifying bioactive compounds through this new screening strategy is based on the results of bioassays, we were concerned about causing the correlation bias which indicated that the identified metabolites might have bioactivities based on the results of the PCA and PLS model, and this correlation bias with the activity result may occur during the metabolite profiling process. In order to overcome this limitation, we tried to resolve the bias. With this purpose, we selected a common metabolite profiling strategy that sorts significantly different compounds by various parts of plants. For this reason, we chose *P. orientalis* and analyzed different parts (fruit, branch, leaf) of this to validate the correlation between identified metabolites and bioactivities. Among 50 plant extracts, the fruit of *P. orientalis* showed high bioactivities in both NO inhibition and antioxidant assays. Additionally, as shown in Figure 1, it was located in the middle of superior NO inhibition and antioxidant extracts. These results indicated that their metabolites could correlate to not only NO inhibition but also antioxidant activities. Hence, we expected that we could validate bioactive compounds obtained from the above correlation analysis by profiling the metabolites from different parts of *P. orientalis*.

### 2.4. Bioactivity Correlations with the Secondary Metabolties of P. orientalis for the Validation of Bioactive Compounds in Indigenous Korean Plants

**Figure 6:**
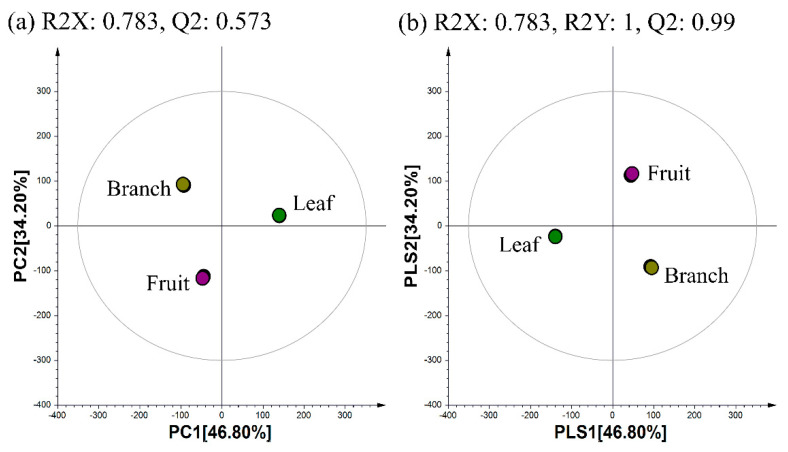
(**a**) PCA and (**b**) PLS-DA score plots of the results obtained for the different parts from *P. orientalis* analyzed by UHPLC-LTQ-Orbitrap-MS/MS. The samples were analyzed using three analytical replicates for each sample. ●: Fruits of *P. orientalis*, ●: Leaves of *P. orientalis*, ●: Branches of *P. orientalis.*

**Figure 7:**
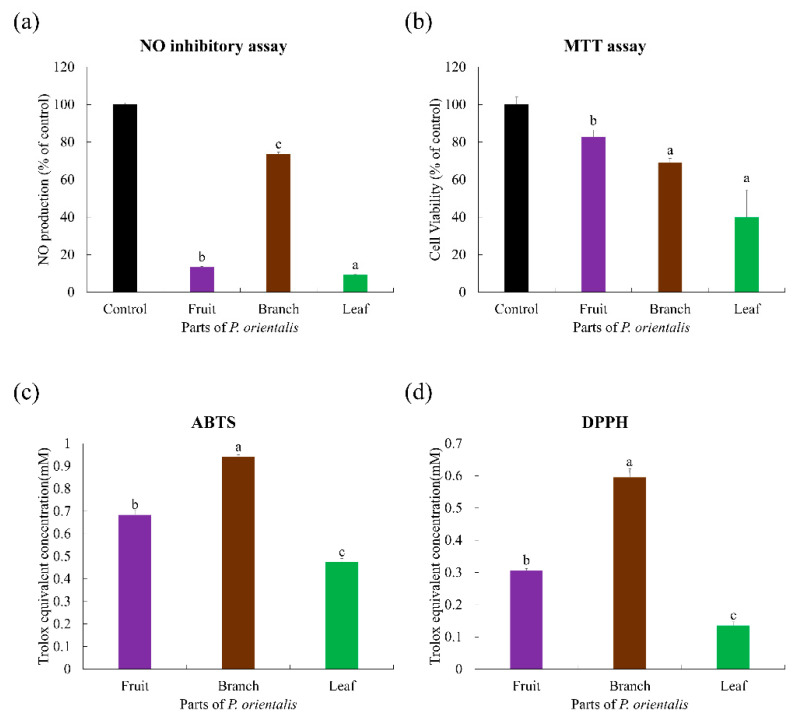
Results of the (**a**) NO inhibitory assay, (**b**) MTT assay, and the antioxidant activity assays, namely (**c**) ABTS and (**d**) DPPH of the extracts of the different parts from *P. orientalis*. Values of (**a**,**b**) are expressed as the mean ± standard deviation (SD) of four biological replicates. Values of (**c**,**d**) are expressed with three biological replicates. Bar graph denoted by the same letter were not significantly different according to Duncan’s multiple range test (*p* < 0.05). Control of (**a**,**b**) indicates LPS alone without treating plant extracts.

Multivariate statistical analyses of the datasets showed distinct metabolomic patterns in the PCA (Figure 6a) and partial least squares-discriminant analysis (PLS-DA; Figure 6b) models. The PCA plot derived from the UHPLC-LTQ-Orbitrap-MS/MS dataset showed that the metabolite profiles based on different parts were separated by PC1 (41.84%) and PC2 (36.45%) (Figure 6a). Similar to the PCA results, the PLS-DA score plot (Figure 6b) could also be readily divided into three groups corresponding to the parts of the samples, along PLS1 (41.84%) and PLS2 (36.45%). Twenty-two metabolites were selected as discriminant metabolites from different parts with VIP > 1.0 and *p*-value < 0.05 obtained from the PLS-DA model (Table S5). The results of the MTT assay showed that the fruits of the plant had high NO inhibitory activity (Figure 7a,b). However, in the antioxidant assay, the plant’s branches showed the highest antioxidant activity among the three tested parts (Figure 7c,d).

**Figure 8:**
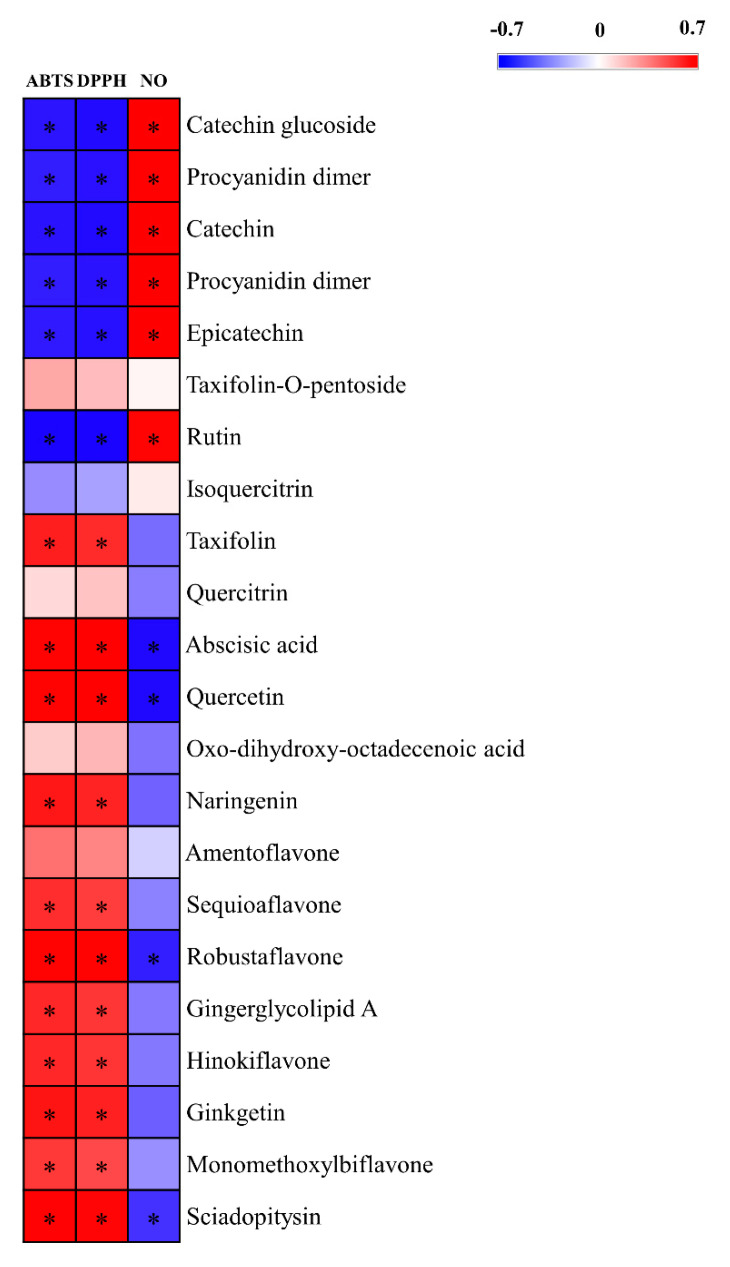
Correlation map between the relative abundance of the significantly different metabolites and the results of the antioxidant assay (ABTS and DPPH), and NO inhibition assay of the extracts of the different parts of P. orientalis. Each square indicates Pearson’s coefficient values (r). Red and blue represent positive (0 < r < 1.0) and negative (−1.0 < r < 0) correlations, respectively. *: *p*-value < 0.05.

Pearson’s correlation analysis tentatively identified compounds that contributed to the observed biological activities of the plant extracts (Figure 8). Four metabolites significantly contributed to NO inhibitory activity, while 11 metabolites were positively correlated with antioxidant activity. Intriguingly, unlike the bioactive compounds in the 50 samples, biflavonoids such as robustaflavone and sciadopitysin contributed to both NO inhibitory and antioxidant activities. Similar to our results, biflavonoids showed antioxidant activity in various studies [56]. Furthermore, Carrillo-Hormaza et al. [57] revealed that biflavonoid content is responsible for the high antioxidant activity of plants and that the biflavonoids were more active than the flavonoid monomers. Thus, through the metabolite profiling of the different parts of *P. orientalis*, we validated the bioactive compounds in the extracts of 50 indigenous plants. In this profiling method, unlike with the above-mentioned process that was based on the results of the bioactivity analysis of different plants, we used differences in the bioactivities of the different parts of *P. oreintalis* to sort out significantly different metabolites without correlation bias. With this method, we revealed that biflavonoids have not only NO inhibitory but also antioxidant activities.

In summary, in screening 50 plant extracts, biflavonoids showed correlation with NO inhibitory activities and phenolic compounds correlated with antioxidant activities, whereas in a validation process using three different parts of *P. orientalis*, biflavonoids showed correlation with these two bioactivities. In this work, we show that the bioactive compound that correlated with bioactivity could be different depending on the method of sorting variable and metabolite profiling, for example based on bioassay results and different parts of plant. Additionally, we reveal that the metabolite obtained using this screening strategy could be used as chemical markers that are responsible for its strong NO inhibitory activity and antioxidant activities. These results pave the way to isolate specific compounds with commercially valuable bioactive properties using appropriate plants on the basis of their respective therapeutic values.

## 3. Materials and Methods

### 3.1. Chemicals and Reagents

High-performance liquid chromatography (HPLC)-grade ethanol, methanol, acetonitrile, and water were purchased from Fisher Scientific (Pittsburgh, PA, USA). All analytical-grade reagents used in this study were obtained from Sigma Chemical Co. (St. Louis, MO, USA).

### 3.2. Plant Materials

Fifty samples of plant extracts obtained from the Wildlife Natural Products Bank (NIBR, Incheon, Korea) were used in this study (Table 1), and all voucher specimens were deposited at the herbarium of the National Institute of Biological Resources (NIBR, Incheon, Korea). The plant samples were dried under shade, and coarsely ground. Each sample (100 g) was extracted for 72 h with 70% ethanol (1 L). The extract was filtered, and the solvent was concentrated using a rotary evaporator (HS-3005W; Hahnshin, Korea). The concentrated extract was further lyophilized, yielding 12% and stored below −20 °C before distribution.

### 3.3. UHPLC-LTQ-Orbitrap-MS/MS Analysis

For UHPLC-LTQ-Orbitrap-MS/MS analysis, each sample (10 mg/mL) was dissolved in 80% ethanol and used. The analysis was performed using a UHPLC system equipped with a Vanquish binary pump H system (Thermo Fisher Scientific, Waltham, MA, USA) coupled with an auto-sampler and column compartment. Chromatographic separation was performed on a Phenomenex KINETEX^®^ C18 Column (100 mm × 2.1 mm, 1.7 μm; Torrance, CA, USA), and the operational parameters were adapted from a study by Lee et al. [25]. The samples were analyzed using three analytical replicates for each sample. To circumvent systematic errors during analysis, the samples were analyzed in random blocks of 10 runs, followed by an intermittent QC sample prepared from pooled blends of each sample extracts. The analytical replicates of the QC samples were clustered at the PCA score plot (Figure S3), ensuring the normal performance of the instrument. The base peak chromatogram of negative and positive mode of QC were shown in Figure S6.

### 3.4. Data Processing and Statistical Analysis

The raw data files from UHPLC-LTQ-Orbitrap-MS/MS were converted into a computable document form (.cdf) format using Thermo Xcalibur v.2.2 (Thermo Fisher Scientific, San Jose, CA, USA). After conversion, MetAlign software (http://www.metalign.nl, accessed on 25 August 2021) was used to preprocess the netCDF data to obtain the baseline correction, peak alignment, peak detection, accurate masses, and normalized peak intensities [48]. The parameters of MetAlign were set according to the specific scaling requirements and chromatographic and mass spectrometric conditions used in the experiments (Tables S1 and S4). Subsequent data, which contained the sample name and peak area information as variables, were transferred to an Excel spreadsheet, and multivariate statistical analyses were performed using the SIMCA-P+ 12.0 software (Umetrics, Umea, Sweden). Both unsupervised PCA and supervised PLS-DA were performed to compare the different metabolites of the samples. PLS-DA was used to improve data interpretability by selecting the influential variables. Based on this, the significant discriminant metabolites were selected uniformly at VIP > 1.0 and *p*-value < 0.05. Cross-validation analysis of the PLS-DA results is summarized in Figure 2. This analysis indicates the prediction accuracy, fitness, and quality of the model. The selected metabolites were identified by comparing their retention times and mass fragment patterns with in-house library data, references, and several databases, such as the National Institutes of Standards and Technology Library (v.2.0, 2011; FairCom, Gaithersburg, MD, USA), the Dictionary of Natural Products (v.16:2, 2007; Chapman and Hall, USA), Wiley 8, and the Human Metabolome Database (HMDB; http://www.hmdb.ca/, accessed on 25 August 2021). Differences in the results of the bioactivity assays were tested by the analysis of variance and Duncan’s multiple range test using PASW Statistics 18 (SPSS Inc., Chicago, IL, USA). Correlations between the metabolites and the bioactivity assay results were calculated via Pearson’s correlation coefficient test using PASW Statistics 18.

### 3.5. Antioxidant Assays

ABTS and DPPH radical scavenging assays were performed to measure the in vitro antioxidant activities of 50 samples of indigenous plants (10 mg/mL of 80% ethanol), following the procedure reported by Lee et al. [58]. All experiments were carried out in triplicate.

### 3.6. Nitric Oxide (NO) Inhibition Assays

RAW 264.7 cells (Code No.: TIB-71, the American Type Culture Collection (Rockville, MD, USA)) were seeded in 96-well plates at a density of 1 × 10^6^ cells/mL in Roswell Park Memorial Institute (RPMI) 1640 supplemented with 100 U/mL penicillin, 100 μg/mL streptomycin, and 10% fetal bovine serum. The cells were grown at 37 °C and 5% CO_2_ in humidified air. After preincubation for 18 h, an LPS stock solution (2 mg/mL) was diluted to 1 µg/mL and administered to the control and sample groups. The plant extracts and control (DMSO in media) with lipopolysaccharide (1 µg/mL) were incubated for 24 h under the same conditions. The nitrite in the culture supernatants was measured by adding 100 μL Griess reagent (1% sulfanilamide and 0.1% *N*- (1-naphthyl)-ethylenediamine dihydrochloride in 5% phosphoric acid) to 100 μL of the supernatant of each sample for 10 min at room temperature. The optical density at 540 nm (OD_540_) was measured using a Spectramax 250 microplate reader (Molecular Devices, Sunnyvale, CA, USA). The assay results were expressed as the mean value of four biological replicates.

### 3.7. MTT Assays for Measuring Cell Viability

Cell viability was measured using the conventional 3- (4,5-dimethylthiazol,2-yl)-2,5-diphenyltetrazolium bromide (MTT) assay with RAW264.7 cells. At 4 h prior to culture termination, 10 μL of MTT solution (10 mg/mL in phosphate-buffered saline) was continuously cultured until termination. Culture was stopped by the addition of 15% sodium dodecyl sulfate to each well to solubilize the formazan produced, and the OD at 570 nm (OD_570-630_) was measured using a microplate Spectramax 250 microplate reader.

The cytotoxicity of the 50 samples was evaluated by the MTT assay. The cell suspension (0.5 × 10^6^ cells/mL) was plated in a 96-well plate. After 2 h of culture, varying concentrations of plant samples were added to each well and cultured for another 6, 24, and 48 h. Cell viability was measured using the MTT assay in four biological replicates.

## 4. Conclusions

In the present study, we used a new multi-parallel metabolomic-cum-bioassay-guided approach to explore the bioactive compounds in 50 indigenous Korean plant extracts. Additionally, we validated these bioactive compounds in different parts of *P. orientalis* to solve the correlation bias of the new screening strategy. From these results, we propose a potential bioactive compound with NO inhibitory and antioxidant activities. Biflavonoids were found to be the major metabolites that contributed to the NO inhibitory activity. Phenolic acids were also positively correlated with antioxidant activity. In the validation step, biflavonoids were found to contribute to both NO inhibitory and antioxidant activities. These results indicate that this validation solved the correlation bias that may occur with the new screening strategy. To the best of our knowledge, this is the first report using a multi-parallel metabolomic-cum-bioassay-guided approach to identify bioactive compounds in 50 indigenous Korean plant extracts. This study showed that metabolite profiling can be performed in line with the purpose of the study or the viewpoints, and as a result, significantly different metabolites can be selected accordingly. Additionally, this study suggested that use of novel screening strategies could be useful for choosing specific plant species and bioactive compounds that contribute to the plant’s bioactivity when profiling various species. Our work and its future applications can help to develop novel as well as culturally relevant plant-based therapeutic approaches. However, a limitation of the present study is that the bioactivities of functional metabolites which showed correlation in this screening has not been performed. Therefore, it is necessary to evaluate their bioactivities and compare each other for further applications.

## Figures and Tables

**Table 1:** Information about the plant samples used in this study.

No.	Labeling	Family	Species	Parts	NIBR Number
1	Lar1	Lardizabalaceae	*Akebia quinata*	stem	NIBRGR0000433376
2	Lar2	Lardizabalaceae	*Akebia quinata*	fruit	NIBRGR0000594502
3	Ama1	Amaryllidaceae	*Allium tuberosum*	etc.	NIBRGR0000612270
4	Ama2	Amaryllidaceae	*Allium tuberosum*	leaf, stem	NIBRGR0000612271
5	Ama3	Amaryllidaceae	*Allium tuberosum*	underground	NIBRGR0000612272
6	Api1	Apiaceae	*Angelica dahurica*	inflorescence	NIBRGR0000612861
7	Ara1	Araliaceae	*Aralia cordata*	stem	NIBRGR0000433525
8	Ara2	Araliaceae	*Aralia cordata*	leaf	NIBRGR0000594470
9	Fag1	Fagaceae	*Castanea crenata*	fruit	NIBRGR0000612236
10	Ran1	Ranunculaceae	*Cimicifuga heracleifolia*	aboveground	NIBRGR0000433787
11	Ran2	Ranunculaceae	*Cimicifuga heracleifolia*	underground	NIBRGR0000611752
12	Com1	Commelinaceae	*Commelina communis*	stem, leaf, flower, fruit	NIBRGR0000594517
13	Ast1	Asteraceae	*Dendranthema boreale*	stem, leaf	NIBRGR0000612853
14	Ast2	Asteraceae	*Dendranthema indicum*	stem, leaf, inflorescence	NIBRGR0000423023
15	Ela1	Elaeagnaceae	*Elaeagnus umbellate*	leaf	NIBRGR0000433417
16	Ela2	Elaeagnaceae	*Elaeagnus umbellate*	branch	NIBRGR0000595364
17	Ara3	Araliaceae	*Eleutherococcus sessiliflorus*	branch	NIBRGR0000433533
18	Ara4	Araliaceae	*Eleutherococcus sessiliflorus*	inflorescence	NIBRGR0000594494
19	Ara5	Araliaceae	*Eleutherococcus sessiliflorus*	leaf	NIBRGR0000597039
20	Cel1	Celastraceae	*Euonymus alatus*	branch	NIBRGR0000595199
21	Lil1	Liliaceae	*Lilium lancifolium*	root	NIBRGR0000597653
22	Lil2	Liliaceae	*Lilium lancifolium*	leaf, stem	NIBRGR0000597655
23	Lil3	Liliaceae	*Lilium lancifolium*	flower	NIBRGR0000610691
24	Lil4	Liliaceae	*Lilium lancifolium*	leaf, stem	NIBRGR0000616820
25	Lau1	Lauraceae	*Lindera obtusiloba*	branch	NIBRGR0000594799
26	Lau2	Lauraceae	*Lindera obtusiloba*	leaf	NIBRGR0000595318
27	Lau3	Lauraceae	*Machilus thunbergia*	branch	NIBRGR0000433461
28	Lau4	Lauraceae	*Machilus thunbergia*	leaf	NIBRGR0000595768
29	Lam1	Lamiaceae	*Mentha arvensis*	aboveground	NIBRGR0000612757
30	Lam2	Lamiaceae	*Mentha arvensis*	underground	NIBRGR0000612758
31	Gra1	Gramineae	*Miscanthus sinensis*	leaf, stem	NIBRGR0000595747
32	Pin1	Pinaceae	*Pinus densiflora*	branch	NIBRGR0000433437
33	Pin2	Pinaceae	*Pinus densiflora*	leaf	NIBRGR0000597148
34	Pla1	Plantaginaceae	*Plantago asiatica*	leaf, peduncle	NIBRGR0000611706
35	Cup1	Cupressaceae	*Platycladus orientalis*	fruit	NIBRGR0000433401
36	Cup2	Cupressaceae	*Platycladus orientalis*	branch	NIBRGR0000433615
37	Cup3	Cupressaceae	*Platycladus orientalis*	leaf	NIBRGR0000597151
38	Sol1	Solanaceae	*Solanum nigrum*	leaf, stem	NIBRGR0000433559
39	Sol2	Solanaceae	*Solanum nigrum*	leaf, stem, inflorescence	NIBRGR0000433569
40	Fab1	Fabaceae	*Sophora flavescens*	leaf	NIBRGR0000594794
41	Fab2	Fabaceae	*Sophora flavescens*	leaf, stem	NIBRGR0000594812
42	Cuc1	Cucurbitaceae	*Trichosanthes kirilowii*	leaf, stem	NIBRGR0000594804
43	Cuc2	Cucurbitaceae	*Trichosanthes kirilowii*	fruit	NIBRGR0000433378
44	Cuc3	Cucurbitaceae	*Trichosanthes kirilowii*	leaf	NIBRGR0000433567
45	Cuc4	Cucurbitaceae	*Trichosanthes kirilowii*	branch	NIBRGR0000433580
46	Typ1	Typhaceae	*Typha orientalis*	leaf, stem	NIBRGR0000595290
47	Typ2	Typhaceae	*Typha orientalis*	inflorescence	NIBRGR0000597721
48	Rut1	Rutaceae	*Zanthoxylum schinifolium*	leaf	NIBRGR0000408953
49	Rut2	Rutaceae	*Zanthoxylum schinifolium*	branch	NIBRGR0000594990
50	Rut3	Rutaceae	*Zanthoxylum schinifolium*	fruit	NIBRGR0000595216

## Data Availability

The data presented in this study are available in supplementary material.

## Supplementary Materials

The following are available online at https://www.mdpi.com/article/10.3390/metabo11090585/s1, Figure S1: (a) NO inhibitory activity and (b) MTT assay. Values are expressed as the average of four biological replicates. Each value is expressed as mean ± SD. The sample information is shown in Table 1, Figure S2: Results of the antioxidant activity assay (a) ABTS, (b) DPPH of the extracts of 50 indigenous plants. Values are expressed as the average of three biological replicates. Each value is expressed as mean ± SD. The sample information are shown in Table 1, Figure S3: PCA score plot derived from positive mode data set of UHPLC-LTQ-Orbitrap-MS/MS of 50 indigenous Korean plant extracts. The quality control (QC) samples were analyzed using five analytical replicates. ▲: 50 indigenous Korean plant extracts, ★: Quality control, Figure S4: UHPLC-LTQ-Orbitrap-MS/MS chromatogram of (a) quality control, (b) C. communis (Com1), (c) E. umbellate (Ela1), (d) E. sessiliflorus (Ara5), (e) P. orientalis (Cup1) and (f) S. flavescens (Fab2). These chromatograms were used to identify metabolites that contributed to NO inhibitory activity, Figure S5: UHPLC-LTQ-Orbitrap-MS/MS chromatogram of (a) quality control, (b) C. crenata (Fag1), (c) E. umbellate (Ela2), (d) M. thunbergia (Lau3), (e) P. orientalis (Cup1) and (f) P. orientalis (Cup2). These chromatograms were used to identify metabolites that contributed to antioxidant activity, Figure S6: Heatmap analysis in 50 Korean indigenous plant extracts derived from UHPLC-LTQ-Orbitrap-MS/MS data. The heatmap indicates the relative contents in secondary metabolites which contribute to NO inhibitory activities. ^a^ Metabolites that have a high contribution to the bioactivities were determined by PLS-biplot (VIP > 1.0, *p* < 0.05). The sample information is shown in Table 1, Figure S7: Heatmap analysis in 50 Korean indigenous plant extracts derived from UHPLC-LTQ-Orbitrap-MS/MS data. The heatmap indicates the relative contents in secondary metabolites which contribute to antioxidant activities. ^a^ Metabolites that have a high contribution to the bioactivities were determined by PLS-biplot (VIP > 1.0, *p* < 0.05). The sample information is shown in Table 1, Table S1: MetAlign settings used to automatically process the experimental dataset of 50 indigenous Korean plant extracts after UHPLC-LTQ-Orbitrap-MS/MS analyses, Table S2: Tentatively identified metabolites from QC samples and 5 plant extracts that contribute to NO inhibitory activityafter UHPLC-LTQ-Orbitrap-MS/MS analyses, Table S3: Tentatively identified metabolites from QC samples and 5 plant extracts that contribute to antioxidant activity after UHPLC-LTQ-Orbitrap-MS/MS analyses, Table S4: MetAlign settings used to automatically process the experimental dataset of *Platycladus orientalis* after UHPLC-LTQ-Orbitrap-MS/MS analyses, Table S5: Discriminant metabolites from three different parts of *Platycladus orientalis* after UHPLC-LTQ-Orbitrap-MS/MS analyses.
