# Supplementary material for: Nontargeted Metabolomics as a Screening Tool for Estimating Bioactive Metabolites in the Extracts of 50 Indigenous Korean Plants
Source: Metabolites. 2021 Aug 30;11(9):585. doi: 10.3390/metabo11090585 (PMC8468114; doi:10.3390/metabo11090585)
Supplement: Supplementary file 1 [file metabolites-11-00585-s001.zip › metabolites-1340905-supplementary/supplemetary information_revision.pdf]

Supplementary Information

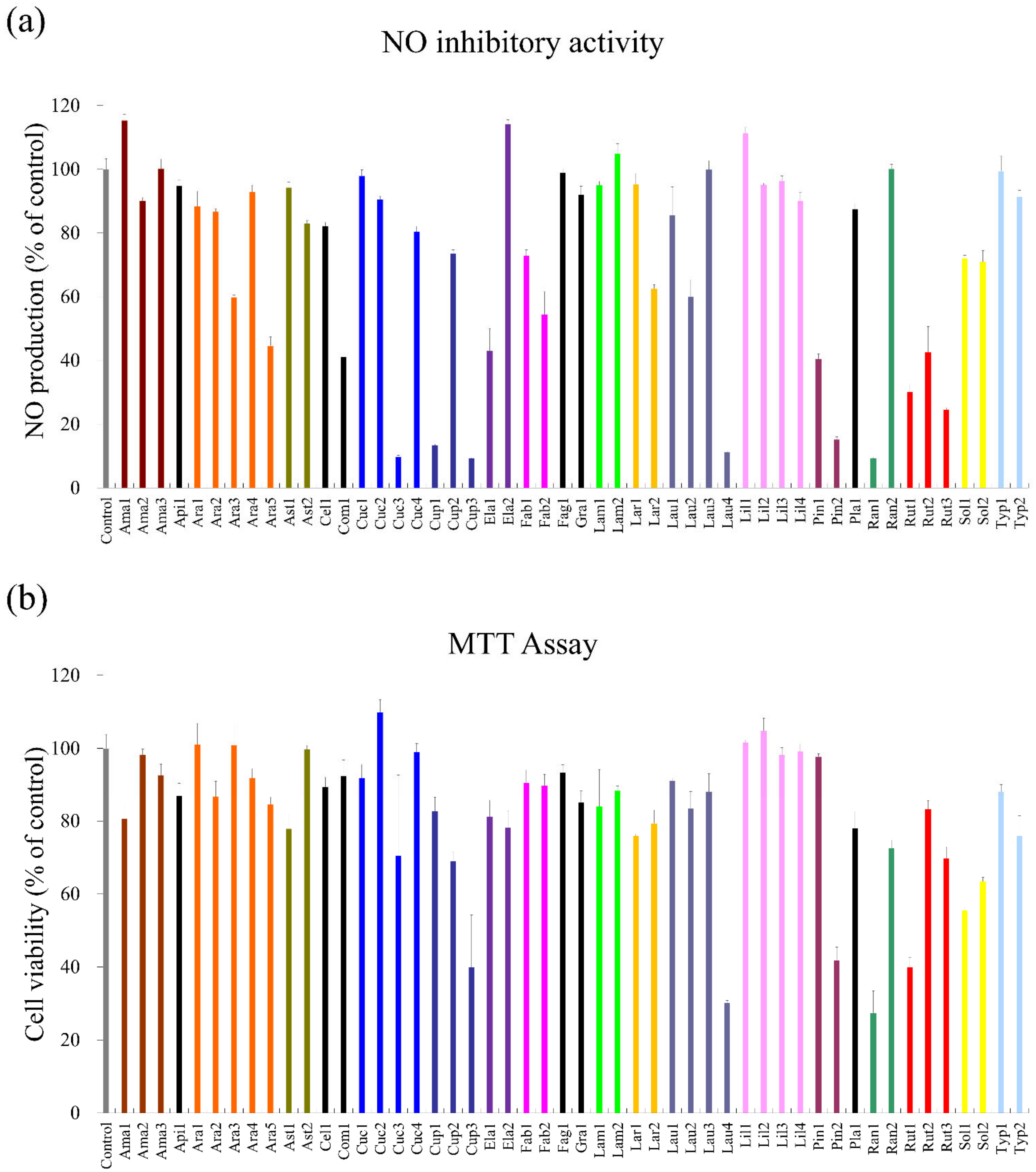

Figure S1. (a) NO inhibitory activity and (b) MTT assay. Values are expressed as the average of four biological replicates. Each value is expressed as mean ± SD. The sample information are shown in Table 1.

(a)

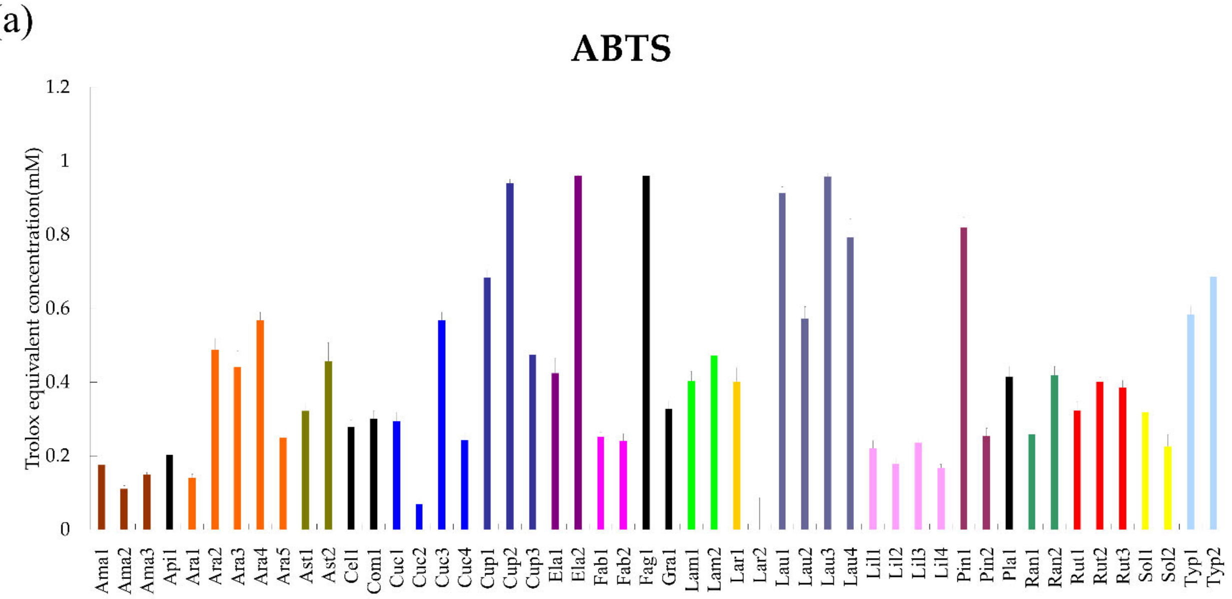

(b)

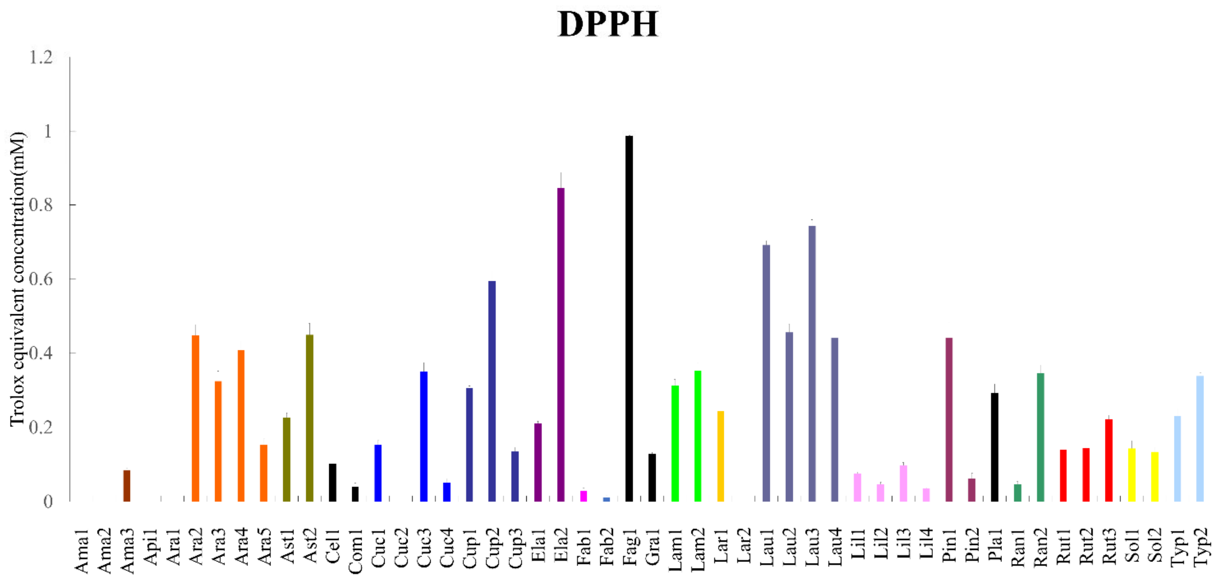

Figure S2. Results of the antioxidant activity assay (a) ABTS, (b) DPPH of the extracts of 50 indigenous plants. Values are expressed as the average of three biological replicates. Each value is expressed as mean ± SD. The sample information are shown in Table 1.

Table S1. MetAlign settings used to automatically process the experimental dataset of 50 indigenous Korean plant extracts after UHPLC-LTQ-Orbitrap-MS/MS analyses.

Parameter	Value
	UHPLC-LTQ-Orbitrap-MS/MS
Retention begin (scan nr.)	1
Retention end (scan nr.)	9600
Maximum amplitude	10,000,000
Peak slope factor (x Noise)	1
Peak threshold factor (x Noise)	2
Peak threshold (Abs. Value)	30
Average peak width at half height (Scans)	90
Scaling Options	None
Maximum shift per scan	30
Select min nr per peak set	9

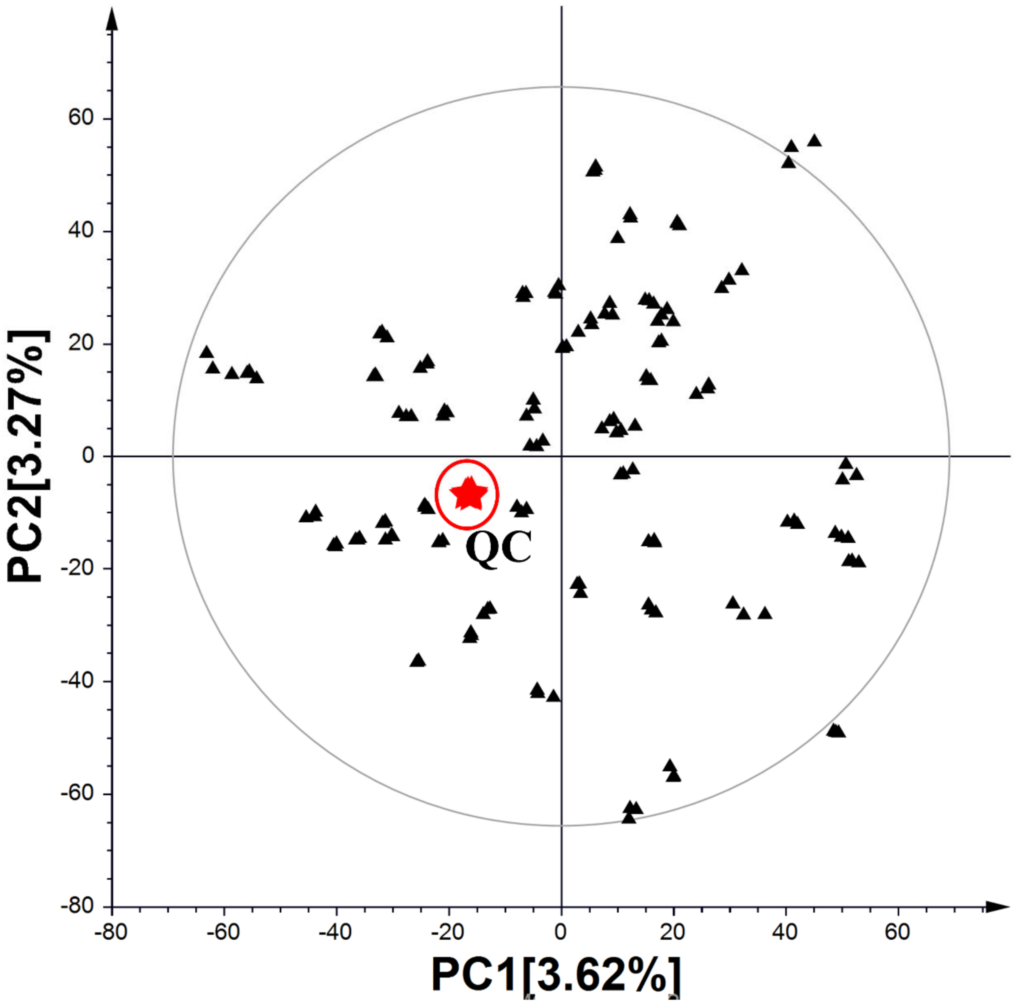

Figure S3. PCA score plot derived from positive mode data set of UHPLC-LTQ-Orbitrap-MS/MS of 50 indigenous Korean plant extracts. The quality control (QC) samples were analyzed using five analytical replicates. ▲: 50 indigenous Korean plant extracts, ★: Quality control

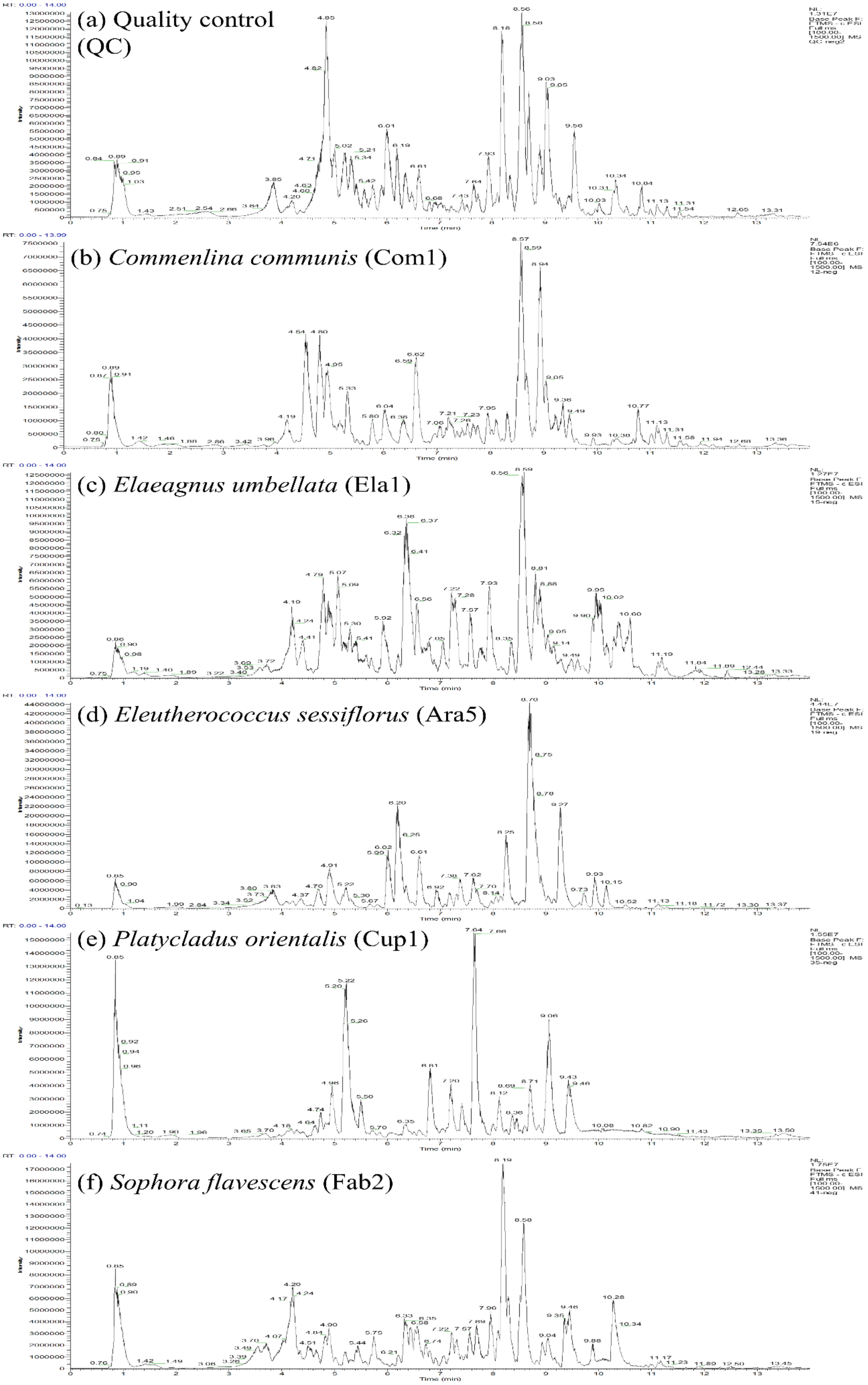

Figure S4. UHPLC-LTQ-Orbitrap-MS/MS chromatogram of (a) quality control, (b) *C. communis* (Com1), (c) *E. umbellata* (Ela1), (d) *E. sessiliflorus* (Ara5), (e) *P. orientalis* (Cup1) and (f) *S. flavescens* (Fab2). These chromatograms were used to identify metabolites that contributed to NO inhibitory activity.

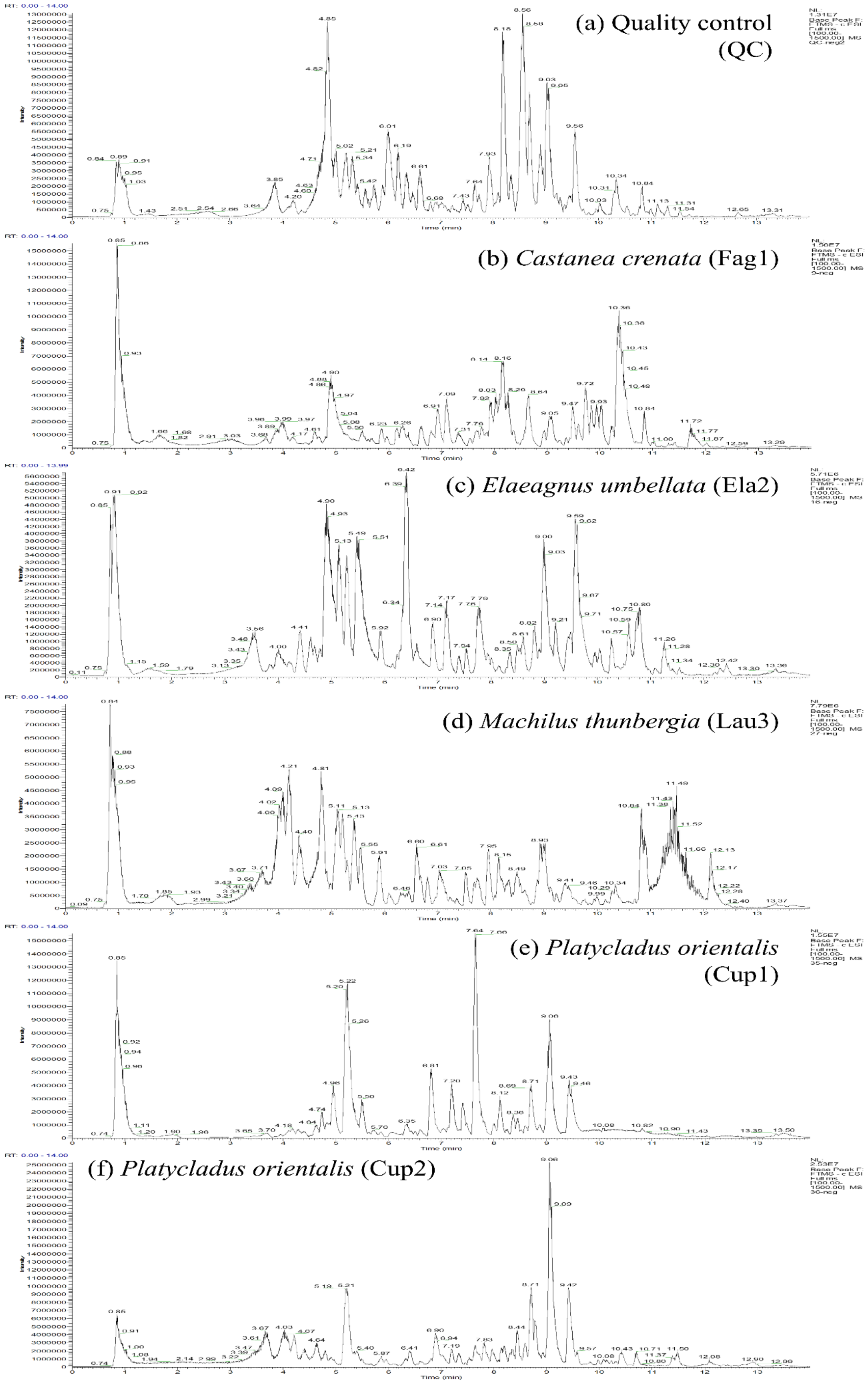

Figure S5. UHPLC-LTQ-Orbitrap-MS/MS chromatogram of (a) quality control, (b) *C. crenata* (Fag1), (c) *E. umbellate* (Ela2), (d) *M. thunbergia* (Lau3), (e) *P. orientalis* (Cup1) and (f) *P. orientalis* (Cup2). These chromatograms were used to identify metabolites that contributed to antioxidant activity.

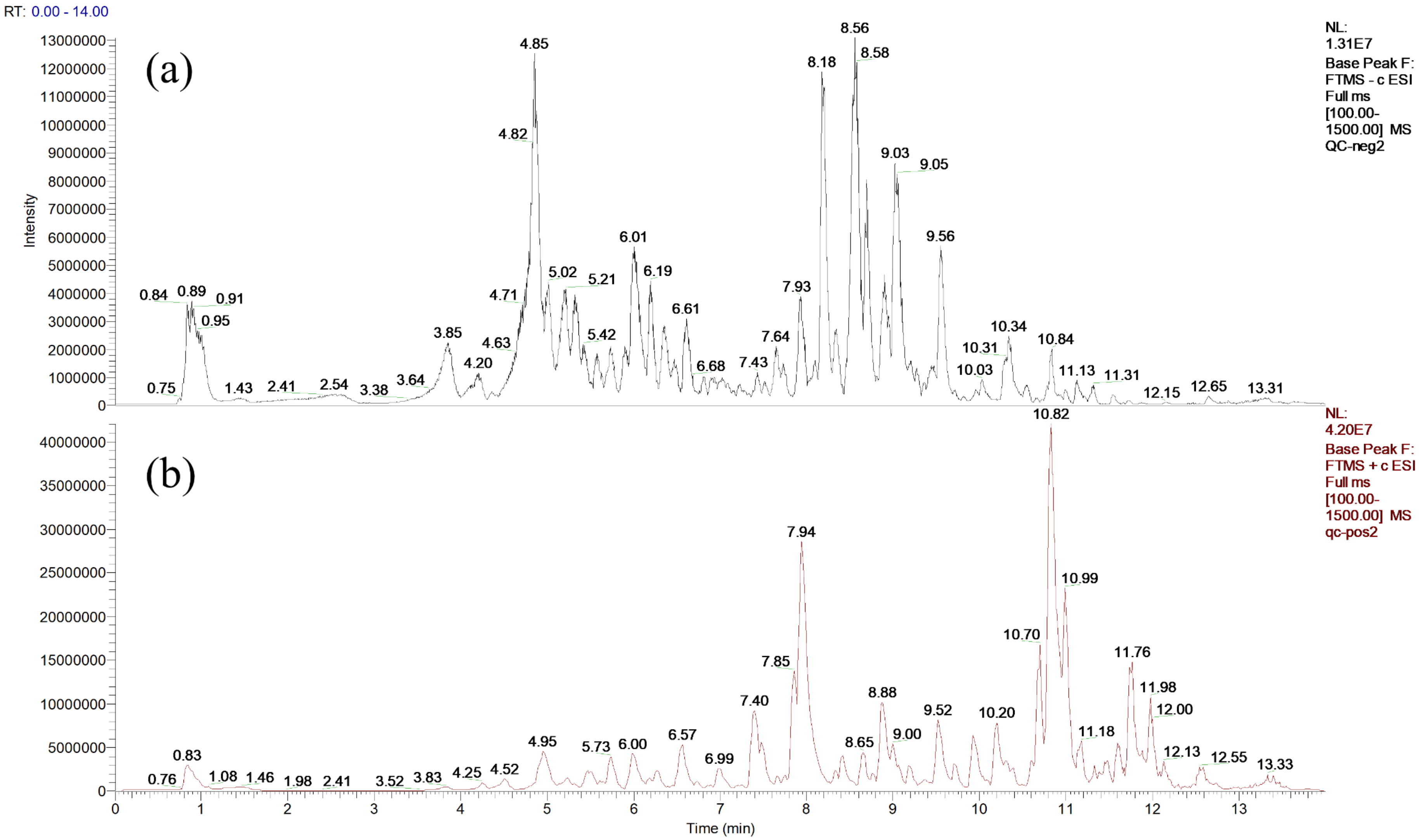

Figure S6. UHPLC-LTQ-Orbitrap-MS/MS chromatogram of (a) negative mode and (b) positive mode of quality control.

Table S2. Tentatively identified metabolites from QC samples and 5 plant extracts that showed high NO inhibitory activity after UHPLC-LTQ-Orbitrap-MS/MS analyses

No.	RT(min)[a]	[M-H]-	[M+H]+	M.W.[b]	Annotation	Molecular formula	Delta ppm[c]	Fragment pattern	VIP1[d]	VIP2	Classification	Annotation levlel*	Ref.[e]
1	0.9	191.0559	215.0536[g]	192	Quinic acid[h]	C7H12O6	-1.2	191>127>85	1.4	1.2	Polyol	2	[1]
2	0.93	387.1135[f]	365.1068[g]	342	Di-caffeoyl acid derivative[h]	C12H22O11	-2.3	387>341>179	1.3	1.2	Phenylpropanoid	3	[2]
3	1.66	353.0868	355.1027	354	3-O-caffeoylquinic acid[h]	C16H18O9	3.2	353>191>126	1.0	1.0	Polyol	3	[3]
4	3.9	865.1976	867.2180	866	Procyanidin trimer[h]	C45H38O18	-0.5	865>695>543>525	3.4	2.8	Biflavonoid	3	[4]
5	4.18	431.1919[f]	409.1858[g]	386	Roseoside	C19H30O8	-1	431>385>153	0.2	0.7	Fatty acyls	3	[5]
6	4.22	289.072	291.0880	290	Epicatechin[h]	C15H14O6	0.2	289>245>203>175	3.6	3.0	Flavonoid	1	[4]
7	4.31	345.1557	369.1541[g]	346	Nepetaside[h]	C16H26O8	-0.5	345>301>161>100	1.2	1.6	Terpenoid	3	[6]
8	4.37	337.0924	339.1097	338	4-*p*-coumaroylquinic acid	C16H18O8	4.7	337>191>127	0.5	0.6	Polyol	3	[7]
9	4.78	609.1448	611.1638	610	Rutin	C27H30O16	-1.6	609>300>271	0.2	0.8	Flavonoid	2	[8]
10	4.96	463.0884	465.1051	464	Isoquercitrin[h]	C21H20O12	0.4	463>316>271>241, 213	1.3	1.1	Flavonoid	3	[8]
11	5.05	593.1500	595.1671	594	Isovitexin 7-O-glucoside	C27H30O15	-0.4	593>311>283	0.4	0.8	Flavonoid	3	[9]
12	5.22	447.0922	449.1096	448	Quercitrin	C21H20O11	-2.4	447>300>271>242	0.9	0.7	Flavonoid	3	[8]
13	5.29	515.1181	517.1365	516	Dicaffeoylquinic acid	C25H24O12	-2.7	515>353>173	0.2	0.6	Polyol	3	[10]
14	6.37	327.2173	351.2161[g]	328	Oxo-dihydroxy-octadecenoic acid	C18H32O5	-3	327>171>127	1.0	0.8	Fatty acid ester	2	[5]
15	6.43	357.1342	359.1506	358	Matairesinol[h]	C20H22O6	0.9	357>313>298>283	1.6	1.3	Lignan	3	[11]
16	6.47	301.0717	303.0878	302	Hesperetin	C16H14O6	3.3	301>286>165	0.6	0.8	Flavonoid	3	[12]
17	6.48	269.0456	271.0605	270	Apigenin	C15H10O5	2.7	269>225>181	0.1	0.9	Flavonoid	3	[13]
18	6.62	329.2329	353.2319[g]	330	Trihydroxy-octadecenoic acid[h]	C18H34O5	-1.4	329>229>211	2.1	2.0	Fatty acid ester	3	[5]
19	6.93	537.0821	539.1013	538	Amentoflavone[h]	C30H18O10	-0.7	537>375>331>287	0.2	1.3	Biflavonoid	3	[14]
20	7.22	307.1909	309.2071	308	Dihydrocapsiate	C18H28O4	2.6	307>289>271>253, 171	0.7	0.9	Phenols	3	[15]
21	7.69	551.0975	553.1149	552	Sequioaflavone[h]	C31H20O10	-1.9	551>389>374>330	0.1	1.1	Biflavonoid	3	[8]
22	7.73	537.0821	539.0986	538	Robustflavone[h]	C30H18O10	-1.3	537>493>465>437, 371	0.6	1.3	Biflavonoid	3	[8]
23	7.97	721.3627[f]	699.3593[g]	676	Gingerglycolipid A	C35H55O14	-1.3	721>675>397	0.3	0.3	Fatty acid ester	3	[16]
24	8.2	565.1136	567.1301	566	Ginkgetin[h]	C32H22O10	-0.3	565>533>518>517	0.9	1.5	Biflavonoid	3	[17]
25	8.66	564.3293[f]	520.3414	519	LysoPC(18:2)	C26H50NO7P	1.9	520>502>443	0.1	0.3	Fatty acid ester	3	[18]
26	8.99	540.3292[f]	496.3435	495	LysoPC(16:0)	C24H50NO7P	2.5	496>478>419	0.4	0.8	Fatty acid ester	2	[18]
27	9.11	579.1293	581.1468	580	Sciadopitysin[h]	C33H24O10	-1.5	579>547>532>503	0.5	1.4	Biflavonoid	3	[19]
28	9.22	566.3454[f]	522.3570	521	LysoPC(18:1)	C26H52NO7P	1.8	522>504>445	0.2	0.4	Fatty acid ester	2	[18]
29	4.95	315.1239	317.1398[g]	316	N.I. (1)[h]	C18H20O5	0.7	315>300>269>251, 147	1.0	1.4			
30	5.07	509.2024	533.2031	510	N.I. (2)[h]	C25H34O11	-0.3	509>491>461>312, 162	1.2	1.5			
31	8.6	653.3727	677.3741[g]	654	N.I. (3)[h]	C31H58O14	-2.8	653>397>235>161	0.4	0.6			

[a] Retention time.
[b] Molecular weight. (g/mol)
[c] Delta ppm based on positive ion mode
[d] Variable importance in projection (VIP > 1.0)
[e] References.
[f] [M+FA-H]-
[g] [M+Na]+
[h] Metabolites that have a high contribution to the bioactivities were selected based on the VIP value (>1.0) and *p*-value (<0.05) from the PLS biplot in Figure 1b.
* {Sumner, 2007 #101}
N.I.: Non-identified metabolite.

Table S3. Tentatively identified metabolites from QC samples and 5 plant extracts that showed high antioxidant activity after UHPLC-LTQ-Orbitrap-MS/MS analyses

NO.	RT(min)[a]	[M-H]$^-$	[M+H]+	M.W.[b]	Annotation	Molecular formula	Delta ppm[c]	Fragment patterns	VIP1[d]	VIP2	Classification	Annotation levlel[*]	Ref.[e]
1	0.90	191.0561	215.0533[g]	192	Quinic acid[h]	C7H12O6	-0.7	191>127>85>57	1.4	1.2	Polyol	2	[1]
2	3.83	353.0872	377.0863[g]	354	Chlorogenic acid	C16H18O9	-1.7	353>191>126>85	0.0	0.5	Polyol	3	[3]
3	3.92	935.0797	937.0978	936	Casuarictin[h]	C41H28O26	-0.5	935>917>873>571	3.0	2.5	Tannin	3	[21]
4	4.18	431.1918[f]	409.1858[g]	386	Roseoside	C19H30O8	1	431>385>153>138	0.2	0.7	Fatty acyls	3	[5]
5	4.21	289.0718	291.0876	290	Epicatechin[h]	C15H14O6	-1.1	289>245>203>175	3.6	3.0	Flavonoid	1	[4]
6	4.28	593.1496	595.1696	594	Vicenin-2	C27H30O15	-1.4	593>473>311>283	0.4	0.5	Flavonoid	3	[22]
7	4.68	433.0416	435.0597	434	Ellagic acid pentoside[h]	C19H14O12	0.3	433>301>256>185	3.4	2.9	Tannin	3	[23]
8	4.84	431.0976	433.1146	432	Isovitexin	C21H20O10	-3.1	431>311>283>239	0.1	0.1	Isoflavonoid	3	[9]
9	4.92	477.0669	479.0837	478	Quercetin 3-O-glucuronide[h]	C21H18O13	-4.1	477>301>178>150	1.0	0.9	Flavonoid	3	[24]
10	4.92	463.0885	465.1051	464	Quercetin 3-O-glucoside[h]	C21H20O12	-3.4	463>301>178>151	1.3	1.1	Flavonoid	3	[24]
11	5.13	433.0773	435.0944	434	Quercetin O-pentoside[h]	C20H18O11	-1	433>301>271>243, 227	2.8	2.2	Flavonoid	3	[24]
12	5.18	447.0938	449.1098	448	Quercitrin	C21H20O11	-2.4	447>300>271>242	0.9	0.7	Flavonoid	3	[8]
13	5.24	431.0976	433.1149	432	Genistein 7-O-glucoside	C21H20O10	-1.6	431>269>225>182	0.1	0.1	Isoflavonoid	3	[25]
14	5.29	515.1181	517.1365	516	Di-O-caffeoylquinic acid	C25H24O12	-2.2	515>353>173>92	0.2	0.6	Phenolic compound	3	[10]
15	5.49	315.0145	317.0305	316	3-O-Methylellagic acid[h]	C15H8O8	-0.5	315>300>243>216, 200	2.8	2.5	Lignan	3	[7]
16	5.95	593.1295	595.1472	594	Kaempferol 3-(p-coumaroyl-glucoside)[h]	C30H26O13	0.6	593>285>256>229	1.8	1.6	Flavonoid	2	[26]
17	6.01	285.0398	287.0563	286	Luteolin	C15H10O6	-2.2	285>241>197>151	0.3	0.8	Flavonoid	3	[27]
18	6.24	953.4717	955.4889	954	Chiisanoside	C48H74O19	-4.4	953>483>441>367	0.1	0.1	Triterpenoid	3	[28]
19	6.43	357.1342	359.1506	358	Matairesinol[h]	C20H22O6	0.9	357>313>298>283	1.6	1.3	Lignan	3	[11]
20	6.61	755.1600	757.1739	756	Quercetin-rhamnoside-rhamnoside-hexoside[h]	C39H32O16	-2.3	755>609>463>301	2.1	1.8	Flavonoid	3	[29]
21	6.62	329.2327	353.2311[g]	330	Trihydroxy-octadecenoic acid[h]	C18H34O5	-1.3	329>229>211>183	2.1	2.0	Fatty acyls	2	[5]
22	7.32	781.1754	783.1941	782	Kaempferol-3-O-acetyl-rhamnogalactoside-7-O-rhamnoside[h]	C23H42O29	1.7	781>635>285>257, 150	2.5	2.1	Flavonoid	3	[30]
23	8.17	565.1136	567.1301	566	Ginkgetin[h]	C32H22O10	-0.3	565>533>518>517	0.9	1.5	Biflavonoid	3	[11]
24	8.99	540.3307[f]	496.3418	495	LysoPC(16:0)	C24H50O7NP	-1.8	540>480>255>237	0.4	0.8	Fatty acid ester	3	[18]
25	0.96	293.0992	295.1146	294	N.I. (1)	C10H18N2O8	1	293>203>131>113	0.8	0.9			
26	4.35	525.1975	549.1974[g]	526	N.I. (2)[h]	C25H34O12	0.5	525>345>165>147	2.2	1.8			
27	4.89	300.9984	303.0154	302	N.I. (3)[h]	C14H6O8	-1.9	301>270>227>199	3.3	2.9			
28	5.15	447.0570	449.0736	448	N.I. (4)[h]	C20H16O12	0.2	447>315>300>244	2.6	2.3			

[a] Retention time.
[b] Molecular weight. (g/mol)
[c] Delta ppm based on positive ion mode
[d] Variable importance in projection
[e] References.
[f] $[M+FA-H]^-$
[g] $[M+Na]^+$
[h] Metabolites that have a high contribution to the bioactivities were selected based on the VIP value (>1.0) and *p*-value (<0.05) from the PLS biplot in Figure 1b.
* {Sumner, 2007 #101}
N.I.: Non-identified metabolite.

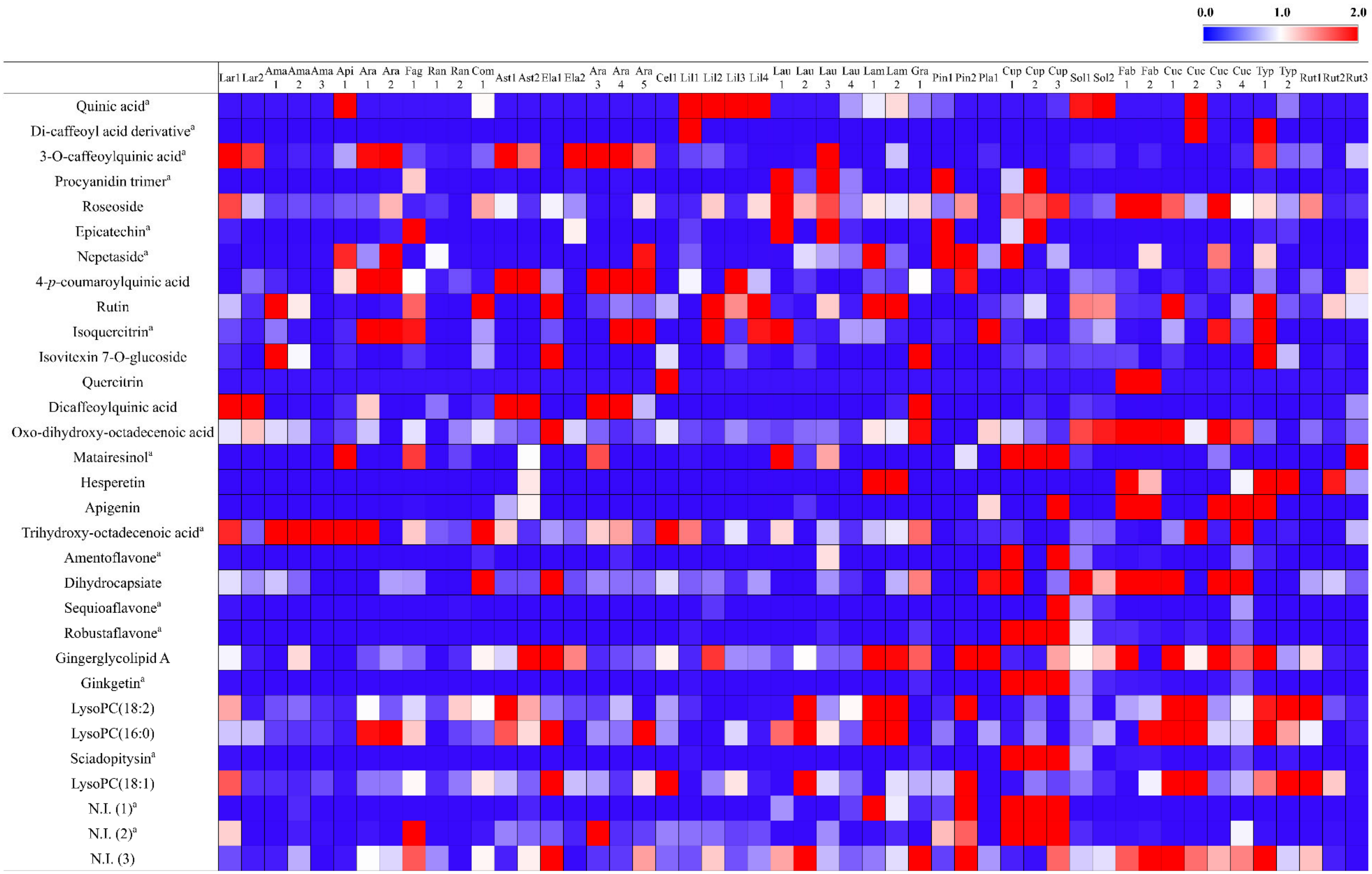

Figure S7. Heatmap analysis in 50 Korean indigenous plant extracts derived from UHPLC-LTQ-Orbitrap-MS/MS data. The heatmap indicates the relative contents in secondary metabolites which contribute to NO inhibitory activities. [a] Metabolites that have a high contribution to the bioactivities were determined by PLS-biplot (VIP > 1.0, $p < 0.05$). The sample information are shown in Table 1. N.I.: Non-identified metabolite.

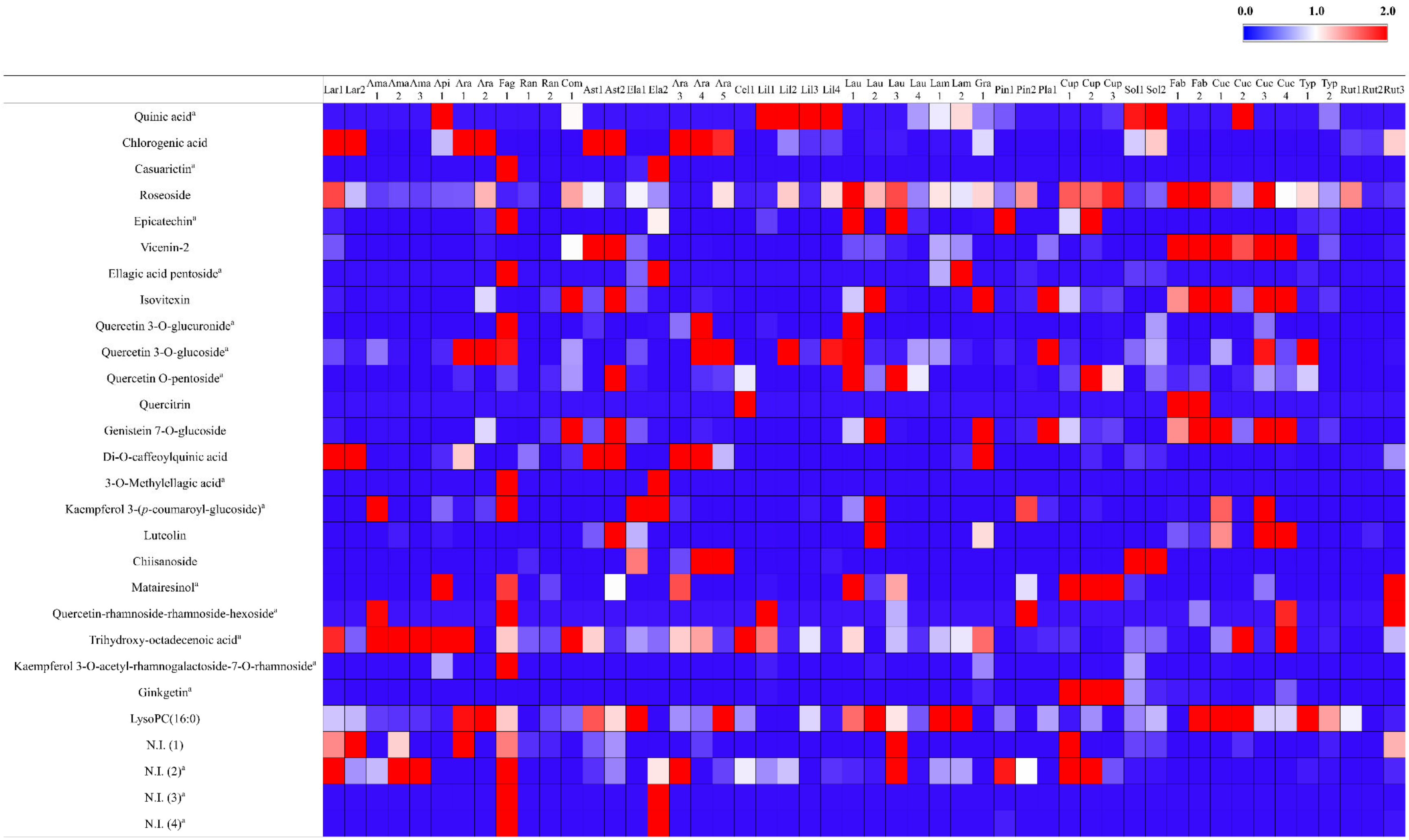

Figure S8. Heatmap analysis in 50 Korean indigenous plant extracts derived from UHPLC-LTQ-Orbitrap-MS/MS data. The heatmap indicates the relative contents in secondary metabolites which contribute to antioxidant activities. [a] Metabolites that have a high contribution to the bioactivities were determined by PLS-biplot (VIP > 1.0, $p < 0.05$). The sample information are shown in Table 1. N.I.: Non-identified metabolite.

Table S4. MetAlign settings used to automatically process the experimental dataset of *Platycladus orientalis* after UHPLC-LTQ-Orbitrap-MS/MS analyses.

Parameter	Value
	**UHPLC-LTQ-Orbitrap-MS/MS**
Retention begin (scan nr.)	1
Retention end (scan nr.)	9600
Maximum amplitude	10,000,000
Peak slope factor (x Noise)	1
Peak threshold factor (x Noise)	2
Peak threshold (Abs. Value)	30
Average peak width at half height (Scans)	40
Scaling Options	None
Maximum shift per scan	40
Select min nr per peak set	9

Table S5. Discriminant metabolites from three different parts of *P. orientalis* samples identified using UHPLC-LTQ-Orbitrap-MS/MS

No.	RT(min)[a]	$[M-H]^-$	$[M+H]^+$	M.W.[b]	Annotation	Molecular formula	Delta ppm.[c]	Fragment pattern	VIP1[d]	VIP2[d]	Annotation levlel[*]	Ref.[e]
1	1.63	451.1242	453.1399	452	Catechin glucoside	C21H24O11	0.0	451>289>245>203	1.0	1.1	3	[31]
2	3.61	577.1355	579.1503	578	Procyanidin dimer	C30H26O12	-1.4	577>425>407>389, 285	1.0	1.1	3	[4]
3	3.65	289.0719	291.0867	290	Catechin	C15H14O6	0.2	289>245>230>175	1.0	1.1	1	[4]
4	4.09	577.1345	579.1502	578	Procyanidin dimer	C30H26O12	-0.9	577>425>407>389, 285	1.0	1.1	3	[4]
5	4.22	289.0721	291.0867	290	Epicatechin	C15H14O6	-0.6	289>245>230>175	1.0	1.1	1	[4]
6	4.67	435.0935	459.0906[g]	436	Taxifolin-O-pentoside	C20H20O11	1.0	435>303, 285>241>199	1.3	1.1	3	[32]
7	4.82	609.1466	611.1619	610	Rutin	C27H30O16	-0.8	609>301>178>161	1.2	1.1	2	[8]
8	4.91	463.0884	465.1036	464	Isoquercitrin	C21H20O12	1.4	459>316>271>241, 214	1.2	1.1	3	[8]
9	5.01	303.0514	305.0662	304	Taxifolin	C15H12O7	0.4	303>285>241>199	1.4	1.0	2	[32]
10	5.23	447.0931	449.1085	448	Quercitrin	C21H20O11	-3.2	447>301>271, 178>151	1.3	1.1	3	[8]
11	5.84	263.1291	287.1260[g]	264	Abscisic acid	C15H20O4	0.6	263>219, 153>137>122	1.5	1.0	3	[33]
12	6.06	301.0357	313.0504[g]	302	Quercetin	C15H10O7	1.2	301>178>150>106	1.5	1.0	2	[8]
13	6.34	327.2181	351.2151[g]	328	Oxo-dihydroxy-octadecenoic acid	C18H32O5	2.7	327>291, 229, 171>153, 127>123	0.3	1.2	2	[5]
14	6.38	271.0614	273.0762	272	Naringenin	C15H12O5	1.1	271>151>107>65	1.4	1.0	2	[34]
15	6.99	537.0833	539.0977	538	Amentoflavone	C30H18O10	1.6	537>443, 417, 375>331>287	1.3	1.1	3	[14]
16	7.70	551.0988	553.1152	552	Sequioaflavone	C31H20O10	0.7	551>457, 389>374, 345>330	1.4	1.0	3	[8]
17	7.76	537.0833	539.0967	538	Robustaflavone	C30H18O10	-0.1	537>375>331>287, 221	1.3	1.1	3	[8]
18	7.94	721.3637[f]	699.3566[g]	676	Gingerglycolipid A	C33H56O14	-1.1	721>675>415, 397>305, 235	1.4	1.0	3	[16]
19	8.08	537.0839	539.0975	538	Hinokiflavone	C30H18O10	-0.3	537>491, 417, 375>347, 331>287	1.4	1.0	3	[8]
20	8.15	565.1133	567.1281	566	Ginkgetin	C32H22O10	1.0	565>533>518>517, 473	1.4	1.0	3	[17]
21	8.31	551.0985	553.1129	552	Monomethoxylbiflavone	C31H20O10	0.8	551>536>492>448	1.4	1.1	3	[8]
22	9.10	579.1296	581.1440	580	Sciadopitysin	C33H24O10	-2.1	579>547>532>503	1.5	1.0	3	[19]

[a] Retention time.
[b] Molecular weight. (g/mol)
[c] Delta ppm based on positive ion mode
[d] Variable importance in projection
[e] References.
[f] $[M+FA-H]^-$
[g] $[M+Na]^+$
*{Sumner, 2007 #101}

References

1. Nyau, V.; Prakash, S.; Rodrigues, J.; Farrant, J. HPLC-PDA-ESI-MS Identification of Polyphenolic Phytochemicals in Different Market Classes of Common Beans (Phaseolus vulgaris L.). *Int. J. Biochem. Res. Rev* **2015**, *8*, 1-11.
2. Berto, A.; Ribeiro, A.B.; de Souza, N.E.; Fernandes, E.; Chisté, R.C. Bioactive compounds and scavenging capacity of pulp, peel and seed extracts of the Amazonian fruit Quararibea cordata against ROS and RNS. *Food Research International* **2015**, *77*, 236-243.
3. He, W.; Liu, X.; Xu, H.; Gong, Y.; Yuan, F.; Gao, Y. On-line HPLC-ABTS screening and HPLC-DAD-MS/MS identification of free radical scavengers in Gardenia (Gardenia jasminoides Ellis) fruit extracts. *Food Chemistry* **2010**, *123*, 521-528.
4. Bystrom, L.M.; Lewis, B.A.; Brown, D.L.; Rodriguez, E.; Obendorf, R.L. Characterisation of phenolics by LC–UV/Vis, LC–MS/MS and sugars by GC in Melicoccus bijugatus Jacq.'Montgomery'fruits. *Food chemistry* **2008**, *111*, 1017-1024.
5. Llorent-Martínez, E.J.; Zengin, G.; Lobine, D.; Molina-García, L.; Mollica, A.; Mahomoodally, M.F. Phytochemical characterization, in vitro and in silico approaches for three Hypericum species. *New Journal of Chemistry* **2018**, *42*, 5204-5214.
6. Yapasert, R.; Sripanidkulchai, B.; Teerachaisakul, M.; Banchuen, K.; Banjerdpongchai, R. Anticancer effects of a traditional Thai herbal recipe Benja Amarit extracts against human hepatocellular carcinoma and colon cancer cell by targeting apoptosis pathways. *Journal of ethnopharmacology* **2020**, *254*, 112732.
7. Zengin, G.; Llorent-Martínez, E.; Sinan, K.I.; Yıldıztugay, E.; Picot-Allain, C.; Mahomoodally, M.F. Chemical profiling of Centaurea bornmuelleri Hausskn. aerial parts by HPLC-MS/MS and their pharmaceutical effects: From nature to novel perspectives. *Journal of pharmaceutical and biomedical analysis* **2019**, *174*, 406-413.
8. Zhuang, B.; Bi, Z.-M.; Wang, Z.-Y.; Duan, L.; Liu, E.-H. Chemical profiling and quantitation of bioactive compounds in Platycladi Cacumen by UPLC-Q-TOF-MS/MS and UPLC-DAD. *Journal of pharmaceutical and biomedical analysis* **2018**, *154*, 207-215.
9. Piasecka, A.; Sawikowska, A.; Krajewski, P.; Kachlicki, P. Combined mass spectrometric and chromatographic methods for in-depth analysis of phenolic secondary metabolites in barley leaves. *Journal of Mass Spectrometry* **2015**, *50*, 513-532.
10. Schütz, K.; Kammerer, D.; Carle, R.; Schieber, A. Identification and quantification of caffeoylquinic acids and flavonoids from artichoke (Cynara scolymus L.) heads, juice, and pomace by HPLC-DAD-ESI/MS n. *Journal of agricultural and food chemistry* **2004**, *52*, 4090-4096.
11. Guo, H.; Liu, A.H.; Ye, M.; Yang, M.; Guo, D.A. Characterization of phenolic compounds in the fruits of Forsythia suspensa by high-performance liquid chromatography coupled with electrospray ionization tandem mass spectrometry. *Rapid Communications in Mass Spectrometry: An International Journal Devoted to the Rapid Dissemination of Up-to-the-Minute Research in Mass Spectrometry* **2007**, *21*, 715-729.
12. Ye, X.; Cao, D.; Zhao, X.; Song, F.; Huang, Q.; Fan, G.; Wu, F. Chemical fingerprint and metabolic profile analysis of Citrus reticulate 'Chachi'decoction by HPLC-PDA-IT-MSn and HPLC-Quadrupole-Orbitrap-MS method. *Journal of Chromatography B* **2014**, *970*, 108-120.
13. Farooq, M.U.; Mumtaz, M.W.; Mukhtar, H.; Rashid, U.; Akhtar, M.T.; Raza, S.A.; Nadeem, M. UHPLC-QTOF-MS/MS based phytochemical characterization and anti-hyperglycemic prospective of hydro-ethanolic leaf extract of Butea monosperma. *Scientific reports* **2020**, *10*, 1-14.
14. Gan, L.; Ma, J.; You, G.; Mai, J.; Wang, Z.; Yang, R.; Xie, C.; Fei, J.; Tang, L.; Zhao, J. Glucuronidation and its effect on the bioactivity of amentoflavone, a biflavonoid from Ginkgo biloba leaves. *Journal of Pharmacy and Pharmacology* **2021**, *73*, 1-11.
15. Lee, G.M.; Suh, D.H.; Jung, E.S.; Lee, C.H. Metabolomics provides quality characterization of commercial gochujang (fermented pepper paste). *Molecules* **2016**, *21*, 921.
16. Ismail, B.B.; Pu, Y.; Guo, M.; Ma, X.; Liu, D. LC-MS/QTOF identification of phytochemicals and the effects of solvents on phenolic constituents and antioxidant activity of baobab (Adansonia digitata) fruit pulp. *Food chemistry* **2019**, *277*, 279-288.
17. Li, C.; Huang, C.; Lu, T.; Wu, L.; Deng, S.; Yang, R.; Li, J. Tandem mass spectrometric fragmentation behavior of lignans, flavonoids and triterpenoids in Streblus asper. *Rapid Communications in Mass Spectrometry* **2014**, *28*, 2363-2370.
18. Fang, N.; Yu, S.; Badger, T.M. LC-MS/MS analysis of lysophospholipids associated with soy protein isolate. *Journal of agricultural and food chemistry* **2003**, *51*, 6676-6682.
19. Gai, Q.-Y.; Jiao, J.; Wang, X.; Liu, J.; Fu, Y.-J.; Lu, Y.; Wang, Z.-Y.; Xu, X.-J. Simultaneous determination of taxoids and flavonoids in twigs and leaves of three Taxus species by UHPLC-MS/MS. *Journal of Pharmaceutical and Biomedical Analysis* **2020**, *189*, 113456.

20. Falleh, H.; Oueslati, S.; Guyot, S.; Dali, A.B.; Magné, C.; Abdelly, C.; Ksouri, R. LC/ESI-MS/MS characterisation of procyanidins and propelargonidins responsible for the strong antioxidant activity of the edible halophyte Mesembryanthemum edule L. *Food Chemistry* **2011**, *127*, 1732-1738.
21. Quatrin, A.; Pauletto, R.; Maurer, L.; Minuzzi, N.; Nichelle, S.; Carvalho, J.; Junior, M.M.; Rodrigues, E.; Bochi, V.; Emanuelli, T. Characterization and quantification of tannins, flavonols, anthocyanins and matrix-bound polyphenols from jaboticaba fruit peel: A comparison between Myrciaria trunciflora and M. jaboticaba. *Journal of food composition and analysis* **2019**, *78*, 59-74.
22. Beelders, T.; De Beer, D.; Stander, M.A.; Joubert, E. Comprehensive phenolic profiling of Cyclopia genistoides (L.) Vent. by LC-DAD-MS and-MS/MS reveals novel xanthone and benzophenone constituents. *Molecules* **2014**, *19*, 11760-11790.
23. Lachowicz, S.; Oszmiański, J.; Rapak, A.; Ochmian, I. Profile and content of phenolic compounds in leaves, flowers, roots, and stalks of Sanguisorba officinalis L. determined with the LC-DAD-ESI-QTOF-MS/MS analysis and their in vitro antioxidant, antidiabetic, antiproliferative potency. *Pharmaceuticals* **2020**, *13*, 191.
24. Barros, L.; Alves, C.T.; Dueñas, M.; Silva, S.; Oliveira, R.; Carvalho, A.M.; Henriques, M.; Santos-Buelga, C.; Ferreira, I.C. Characterization of phenolic compounds in wild medicinal flowers from Portugal by HPLC–DAD–ESI/MS and evaluation of antifungal properties. *Industrial Crops and Products* **2013**, *44*, 104-110.
25. Stobiecki, M.; Staszków, A.; Piasecka, A.; Garcia-Lopez, P.M.; Zamora-Natera, F.; Kachlicki, P. LC-MSMS profiling of flavonoid conjugates in wild Mexican lupine, Lupinus reflexus. *Journal of Natural Products* **2010**, *73*, 1254-1260.
26. Spínola, V.; Pinto, J.; Llorent-Martínez, E.J.; Castilho, P.C. Changes in the phenolic compositions of Elaeagnus umbellata and Sambucus lanceolata after in vitro gastrointestinal digestion and evaluation of their potential anti-diabetic properties. *Food Research International* **2019**, *122*, 283-294.
27. Desta, K.T.; Kim, G.S.; Abd El-Aty, A.; Raha, S.; Kim, M.-B.; Jeong, J.H.; Warda, M.; Hacımüftüoğlu, A.; Shin, H.-C.; Shim, J.-H. Flavone polyphenols dominate in Thymus schimperi Ronniger: LC–ESI–MS/MS characterization and study of anti-proliferative effects of plant extract on AGS and HepG2 cancer cells. *Journal of Chromatography B* **2017**, *1053*, 1-8.
28. Yang, S.; Chun-Juan, Y.; Kai, Y.; Fa-Mei, L. In vivo antithrombotic and antiplatelet activities of a quantified Acanthopanax sessiliflorus fruit extract. *Chinese Journal of Natural Medicines* **2011**, *9*, 141-145.
29. Molina-García, L.; Martínez-Expósito, R.; Fernández-de Córdova, M.; Llorent-Martínez, E. Determination of the phenolic profile and antioxidant activity of leaves and fruits of Spanish Quercus coccifera. *Journal of Chemistry* **2018**, *2018*.
30. Neugart, S.; Rohn, S.; Schreiner, M. Identification of complex, naturally occurring flavonoid glycosides in Vicia faba and Pisum sativum leaves by HPLC-DAD-ESI-MSn and the genotypic effect on their flavonoid profile. *Food Research International* **2015**, *76*, 114-121.
31. Nyau, V.; Prakash, S.; Rodrigues, J.; Farrant, J. Idenfication of nutraceutical phenolic compounds in bambara groundnuts (Vigna subterranea L. Verdc) by HPLC-PDA-ESI-MS. *Current Journal of Applied Science and Technology* **2015**, 77-85.
32. Mämmelä, P. Phenolics in selected European hardwood species by liquid chromatography–electrospray ionisation mass spectrometryElectronic Supplementary Information available. See http://www. rsc. org/suppdata/an/b1/b104584a. *Analyst* **2001**, *126*, 1535-1538.
33. Durgbanshi, A.; Arbona, V.; Pozo, O.; Miersch, O.; Sancho, J.V.; Gómez-Cadenas, A. Simultaneous determination of multiple phytohormones in plant extracts by liquid chromatography− electrospray tandem mass spectrometry. *Journal of agricultural and food chemistry* **2005**, *53*, 8437-8442.
34. Sun, J.; Liang, F.; Bin, Y.; Li, P.; Duan, C. Screening non-colored phenolics in red wines using liquid chromatography/ultraviolet and mass spectrometry/mass spectrometry libraries. *Molecules* **2007**, *12*, 679-693.
